# Unveiling the Balkans’ advances: *In vitro* biotechnology of woody plants in the early 21^st^ century

**DOI:** 10.3389/fpls.2025.1586013

**Published:** 2025-08-25

**Authors:** Valbona Sota, Lilyana Nacheva, Dejan Bošnjak, Eleni Abraham, Slađana Jevremović, Branislav Cvjetković, Vladislava Galović, Darko Jevremović, Zvjezdana Marković, Efigjeni Kongjika, Sanja Bogunović, Svjetlana Zeljković, Vlatko Andonovski, Vanja Daničić, Tatjana Vujović

**Affiliations:** ^1^ Department of Biotechnology, Faculty of Natural Sciences, University of Tirana, Tirana, Albania; ^2^ Research Center of Biotechnology and Genetics, Academy of Sciences of Albania, Tirana, Albania; ^3^ Fruit Growing Institute, Agricultural Academy, Plovdiv, Bulgaria; ^4^ Faculty of Agrobiotechnical Sciences Osijek, Josip Juraj Strossmayer University of Osijek, Osijek, Croatia; ^5^ Faculty of Agriculture, Forestry and Natural Environment, Aristotle University of Thessaloniki, Thessaloniki, Greece; ^6^ Institute for Biological Research “Siniša Stanković” - National Institute of Republic of Serbia, University of Belgrade, Belgrade, Serbia; ^7^ Faculty of Forestry, University of Banja Luka, Banja Luka, Bosnia and Herzegovina; ^8^ Institute of Lowland Forestry and Environment, University of Novi Sad, Novi Sad, Serbia; ^9^ Fruit Research Institute, Čačak, Serbia; ^10^ Department of Viticulture and Enology, Faculty of Agriculture, University of Zagreb, Zagreb, Croatia; ^11^ Department of Genetics, Forest Tree Breeding and Seed Science, Croatian Forest Research Institute, Jastrebarsko, Croatia; ^12^ Faculty of Agriculture, University of Banja Luka, Banja Luka, Bosnia and Herzegovina; ^13^ Hans Em Faculty of Forest Sciences, Landscape Architecture and Environmental Engineering, University Ss. Cyril and Methodius in Skopje, Skopje, North Macedonia

**Keywords:** Balkan countries, micropropagation, *in vitro* conservation, woody plants, genetic resources

## Abstract

The Balkan Peninsula is a European biodiversity hotspot, home to 6,500 native vascular plant species, many of which are endemic. The region has diverse range of climates and complex topography, creating conditions that suit many woody ornamental, fruit, and forest species. Nevertheless, climate change, habitat destruction, invasive species, plant diseases, and agricultural practices threaten natural ecosystems and cultivated species. Many Balkan countries have addressed these challenges using advanced biotechnological approaches, including micropropagation, *in vitro* conservation, and *in vitro* selection for stress-tolerant genotypes. This paper provides a comprehensive overview of *in vitro* plant biotechnology progress in the Balkan countries from the beginning of the 21^st^ century to the present, with a focus on woody horticultural and forest species. The primary objectives of scientific research include optimizing media composition for all components and micropropagation stages, as well as effective initial explant selection. In addition, temporary immersion bioreactors, synthetic seed technology, and cryopreservation techniques have been explored to enhance plant production and conservation. Scientific research for woody ornamentals and fruit tree species has progressed in Albania, Bulgaria, Croatia, Greece, and Serbia while remaining limited in other Balkan countries. Forest tree production research is currently conducted in Bulgaria, Greece, and Serbia, with labs focusing on micropropagation and *ex situ* conservation. In addition to advances in scientific research, several commercial companies operate in the Balkans, with establishments in Serbia, Greece, Bulgaria, and North Macedonia, that produce *in vitro*-derived planting material for fruit trees and woody ornamental plants. Despite this progress, research in the Balkan countries remains fragmented, emphasizing the need to strengthen regional collaboration and knowledge exchange to promote agricultural development processes and biotechnological applications in this region. This review represents the first exploration of this topic in the Balkans and successfully unites researchers from several countries. It highlights key scientific advances in *in vitro* biotechnology for woody plants, identifies challenges, and proposes solutions. The value of interdisciplinary collaboration is emphasized through the optimization of *in vitro* methodologies, the promotion of germplasm conservation, and the sustainable use of plant genetic resources in the Balkans.

## 1 Introduction

**Figure 1:**
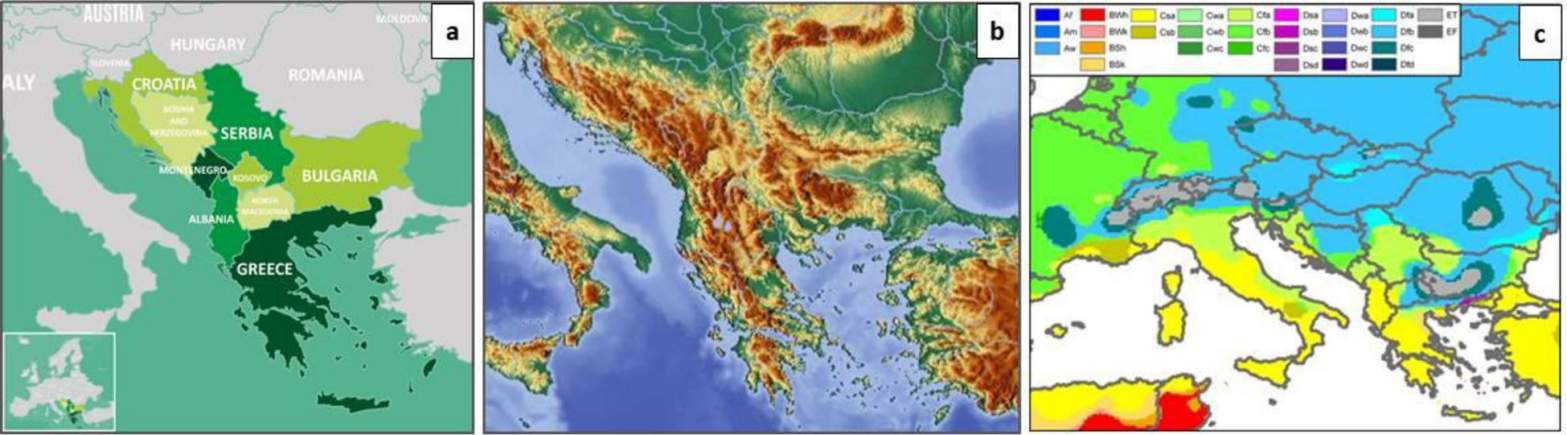
**(a)** The map of the Balkan countries **(b)** Topography of the region **(c)** Köppen-Geiger climate type map of the Balkan Peninsula (Peel et al., 2007).

The Balkan Peninsula, located in southeastern Europe, is bordered by the Adriatic Sea to the west, the Ionian and Mediterranean to the south, and the Black Sea to the east. While its northern boundaries are debated, nine countries—Albania, Bosnia and Herzegovina, Bulgaria, Croatia, Greece, Kosovo^1^, Montenegro, North Macedonia, and Serbia—are generally considered part of the Balkans (**Figure 1a**) (Reed et al., 2004). Other countries, like Slovenia and Romania, have only small portions of land within the peninsula. Despite Turkey’s European territory, it is not typically classified as a Balkan country.

Strategically positioned at the intersection of Europe, Asia, and Africa, the Balkan region has long been a bridge for cultural, social, and economic exchange. This has made the region a key player in European politics and integration efforts. Many Western Balkan countries—Albania, Bosnia and Herzegovina, Kosovo, Montenegro, North Macedonia, and Serbia—are not EU members but are part of EU strategic initiatives that support biodiversity conservation, environmental protection, and agricultural development.

The region’s varied landscape—mountains, valleys, grasslands, lakes, rivers, and coastal areas—creates diverse microclimates and habitats (**Figure 1b**) (Allcock et al., 2024). This climatic and geographic variation is a key reason for the Balkans’ rich biodiversity and endemic plant species. A Mediterranean climate prevails in the south, while the north experiences a Continental climate (**Figure 1c**), resulting in distinct plant communities, contributing to different plant communities. With a history of unique vegetation shaped by geography and climate, the Balkans are considered a biodiversity hotspot in Europe. Nearly a century ago, Turill (1929) noted that this region hosts more plant species than any other comparable part of Europe. Covering just 5.2% of the continent, the Balkans are home to around 6,500 native vascular plant species, many of which are endemic (Polunin, 1980). Relict flora is often found at higher altitudes, with each country contributing uniquely to this diversity:

**– **Albania: 3,976 species, with 32 endemic species and 150 endemic subspecies (Zeneli et al., 2014);
**– **Bosnia and Herzegovina: ~3,572 plant taxa, including ~500 endemics (BIOFOR, 2003);
**– **Bulgaria: 6,275 species, including cultivated and wild flora (Stoyanov et al., 2022);
**– **Croatia: 4,500 species, nearly 50% forming diverse forest ecosystems (Lovrić and Lovrić, 2013);
**– **Greece: 6,620 taxa, with 1,459 (22%) endemic due to its complex terrain and historical influences (Dimopoulos et al., 2016);
**– **Kosovo: Approximately 2800–3000 plant species (Millaku et al., 2013);
**– **Montenegro: 3,250 vascular plant species (Ministry of Spatial Planning and Environment, 2010);
**– **North Macedonia: Nearly 3,700 species, including 120 endemics (Matevski, 2013; Convention on Biological Diversity, 2022);
**– **Serbia: 3,662 vascular plant taxa (39% of Europe’s flora), including 547 Balkan endemics (Šijačić-Nikolić et al., 2014);

The region’s biodiversity also extends to cultivated species. The Balkans produces a wide range of fruits, including pome fruits (apple, pear, quince, medlar, and rowan), stone fruits (peach, nectarine, apricot, plum, cherry, and sour cherry), nut fruit (walnut, hazelnut, and almond), berries (strawberry, raspberry, blackberry, currant, gooseberry, blueberry and kiwifruit), grapevine, and Mediterranean species (olive, mandarin, fig, pomegranate, carob, persimmon, orange, lemon) (Panjković et al., 2021; Hansjörg and Seefeld-Hechendorf, 2022).

However, biodiversity in the Balkans is threatened by habitat destruction, overexploitation of resources, and climate change. Rising temperatures, shifting rainfall patterns, and extreme weather events already affect natural forests and cultivated plants (EEA, 2023). Müller and Hofmann (2022) warn that these challenges will only intensify, placing even greater stress on land and biodiversity. Forests, woody crops, and ornamentals are particularly vulnerable due to their long lifespan and slow adaptation to ecological changes (Giannini and von Wühlisch, 2009). Climate change is also fueling the spread of pests and pathogens.

Traditional agricultural and conservation methods may no longer be sufficient to protect plant biodiversity and maintain healthy crop production. Biotechnological approaches such as micropropagation, *in vitro* conservation, and *in vitro* selection for stress resistance are becoming increasingly important, if not essential. These techniques support genetic diversity, help restore ecosystems, and improve plant production for conservation and commercial purposes (Atanassov and Batchvarova, 2002). For many years, research institutions and universities across the Balkans have been working to refine *in vitro* methods for propagating and conserving forest and fruit trees. However, these efforts have remained mainly isolated within individual countries, making it difficult to access the collective progress made so far. Until now, there has been no comprehensive overview of advancements in *in vitro* techniques applied to woody plants on a Balkan scale.

This review fills this gap by summarizing the current state of *in vitro* woody plant propagation and conservation in the Balkans, highlighting key advancements, challenges, and commercialization potential from 2001 to 2024. Moreover, it contributes to establishing international and multidisciplinary cooperation among Balkan researchers, an essential initiative to strengthen communication and collaboration across borders.

## 2 Research and applications of *in vitro* woody plant biotechnology in the Balkan countries

### 2.1 Leading research institutions in the region

Research in plant tissue culture across the Balkans has evolved over the decades, with some countries pioneering *in vitro* techniques early on, while others have gradually integrated them into academic and research institutions. While certain countries boast well-established labs and active research programs, others are still developing their capabilities.

Albania’s earliest scientific efforts in micropropagation techniques began at the Vegetables and Potato Institute in Tirana (1988–1990). Over time, more labs have been gradually established in universities or research institutions. One of the most influential was the plant tissue culture lab at the Institute of Biological Research, Academy of Sciences of Albania (ASA), led by Professor Efigjeni Kongjika from 1991 to 2008, a lab that played a crucial role in advancing *in vitro* techniques for various plant species (Kongjika et al., 1995). In 2008, the Department of Biotechnology at the University of Tirana was established as part of a structural reform within ASA, incorporating the existing infrastructure and expertise and introducing courses in plant tissue culture. The Agricultural Technology Transfer Center (ATTC) in Vlora also operates a plant tissue lab focused on rootstock micropropagation. In 2024, a dedicated micropropagation facility was established at the Research Center of Biotechnology and Genetics, ASA, to strengthen collaboration through the National Biotechnology Network in Albania.

Bosnia and Herzegovina initiated micropropagation in the early 1990s, particularly in Mostar, where the “Hepok” greenhouse focused on ornamental species, such as carnations. Unfortunately, with the onset of the civil war, the greenhouse ceased operations, and the programs were subsequently closed. Currently, micropropagation is taught in courses at the University of Banja Luka and the University of Sarajevo. The Republic of Srpska has two active labs at the University of Banja Luka—one at the Faculty of Agriculture and another at the Institute for Genetic Resources. Both labs are involved in scientific research, student training, and the preservation of endangered plant species, especially vegetables and herbaceous plants. Recent research has been conducted on cloning some *Prunus* species of interest to expand studies to include other woody plants in Bosnia and Herzegovina.

Bulgaria’s involvement in *in vitro* plant biotechnology began in 1983 with the initiation of a national program aimed at improving horticulture development. The program’s goal was to apply plant biotechnology broadly in the breeding programs of the specialized crop institutes in the country and the promotion of commercialization (Atanassov, 1995). Large-capacity micropropagation labs have been established at several institutes, including the Fruit Growing Institute, the Institute of Vegetable Crops in Plovdiv, the Institute of Plant Gene Resources in Sadovo, and the Institute of Floriculture in Sofia. The Agricultural Academy set up the Institute of Genetic Engineering in 1985 to coordinate research and three commercial labs for the micropropagation of fruit, vegetable, and ornamental species (Yordanov, 1986). Plant biotechnology research is also conducted in institutes organized under the Bulgarian Academy of Sciences, Sofia University, Plovdiv University, the University of Forestry in Sofia, and the Agricultural University in Plovdiv.

Croatia is well known for its *in vitro* research, especially in woody plants. Key institutions include the University of Zagreb, Josip Juraj Strossmayer University in Osijek, the Ruđer Bošković Institute, the Croatian Forest Research Institute, the Agricultural Institute Osijek, and the Institute for Adriatic Crops in Split. The first experiments with *in vitro* fruit species began in the late 1980s at the University of Zagreb. Furthermore, the Ruđer Bošković Institute and the Faculty of Science in Zagreb have been conducting research on forest trees for over 25 years. Research has expanded, with the Croatian Forest Research Institute opening a micropropagation lab and a Plant Tissue Bank in 2018, which is linked to the National Gene Bank. The grapevine tissue culture lab at the University of Zagreb focuses on biotechnology for research and education but is not involved in commercial applications.

Greece conducts plant tissue culture research primarily in agricultural, biology, and forestry schools, with key contributions from academic institutions such as Aristotle University of Thessaloniki (AUTh), Agricultural University of Athens (AUA), and Democritus University of Thrace. Institutions like the Mediterranean Agronomic Institute of Chania (CIHEAM Chania) and the National Institute of Agriculture Research ELGO-DEMETRA also play a significant role. Olives, grapevines, cultivated fruit trees, and other medicinal, aromatic, and decorative plants are the main focus as they are both economically and ecologically significant.

North Macedonia started its *in vitro* research in the early 1980s at the Faculty of Natural Sciences and Mathematics of the University Ss. “Cyril and Methodius” in Skopje, and focuses on improving the production of secondary metabolites in crops, ornamentals, and herbaceous medicinal plants. Later, the University of Goce Delcev in Shtip joined the effort, specializing in vegetables and herbaceous plants. Recent projects at the University Ss. “Cyril and Methodius” aim to optimize micropropagation protocols for woody plants to produce disease-free plants and conserve valuable genetic material.

Serbia has been a pioneer in woody plant cloning for nearly 50 years. Research started in the 1970s at the Institute for Biological Research “Siniša Stanković” - National Institute of the Republic of Serbia, University of Belgrade, (IBISS) with groundbreaking work by Professor Ljiljana Radojević, first on hazelnut (Radojević et al., 1975), and later extending to various endemic conifers and ornamental trees. The Fruit Research Institute (FRI) in Čačak began *in vitro* propagation of fruit species in 1982. It later developed *in vitro* propagation and conservation protocols for numerous pome, stone, and berry fruits. The Institute of Lowland Forestry and Environment (ILFE) in Novi Sad also began cloning other woody species like poplars and black locust, in the 1980s to establish seed and commercial plantations. Later, it focused on stress tolerance in poplars and wild cherry, as well as genome editing in white poplar. Additionally, the Faculty of Agriculture (UNSFA) in Novi Sad has operated the lab for micropropagation since 1983, employing tissue culture techniques for a wide range of horticultural crops, including fruit trees and grapevines. In 2001, a tissue culture lab was also established at the Faculty of Forestry (UB-FF) in Belgrade.

Kosovo and Montenegro have not yet developed any plant tissue culture labs for scientific research, highlighting an opportunity for future development.

### 2.2 Key plant categories and *in vitro* advances from 2001 to 2024

#### 2.2.1 Woody crops and ornamental species

Albania has made significant advancements in *in vitro* plant biotechnology, focusing on autochthonous and cultivated fruit species (**Table 1**, **Supplementary File**, Rows-R). Research efforts have primarily concentrated on the following areas: (i) Optimizing micropropagation protocols: Extensive studies have been conducted to refine basal media composition, plant growth regulator (PGR) concentrations and types, sterilization methods, explant selection, sugar sources, and oxidative stress management. These efforts have targeted numerous species, including kiwifruit (R: 1), myrtle (R: 11–12), pomegranate (R: 15 – 17), GF 677 rootstock (R: 40), apricot (R: 43), wild cherry (R; 45), Gisela 6 rootstock (R: 63), mahaleb cherry (R: 95), jujube (R: 98), apple (R: 99), wild apple (R: 113), wild pear (R: 120), almond-leaved pear (R: 122), walnut (R: 124–125), Albanian forsythia (R: 140), grape (R: 147). (ii) Slow growth *in vitro* conservation: To ensure the preservation of plant genetic resources, various conservation methods have been developed, including low-temperature storage, nutrient media modifications, and controlled dehydration of explants for short- and medium-term storage. These strategies have been successfully applied to myrtle (R: 11), wild cherry (R: 45), jujube (R: 98), wild pear (R: 119), almond-leaved pear (R: 121); (iii) Temporary immersion systems (TIS bioreactors): Systems such as ElecTIS, Plantform™, and SETIS™ have been employed to enhance plant production and quality. These systems have demonstrated improved shoot proliferation and superior plantlet morphology compared to conventional micropropagation techniques in pomegranate (R: 13), plum (R: 76), wild apple (R: 114), and wild pear (R: 120); (iv) Synthetic seed technology: Encapsulation has been investigated to develop efficient protocols for plantlet regrowth under different chemical and physical incubation conditions. Research has focused on pomegranate (R: 18) and walnut (R: 123). Future efforts aim to advance cryopreservation techniques for the long-term conservation of valuable plant genetic resources.

Bulgaria has successfully developed regeneration methods for a wide range of important woody species, and effective protocols have been developed for successful micropropagation of the main Bulgarian raspberry varieties – ‘Bulgarian rubin’, ‘Samodiva’, ‘Shopska alena’, ‘Iskra’ and ‘Lyulin’ (**Table 1**, **Supplementary File**, Rows-R). Reliable micropropagation protocols have been developed for many woody fruit and ornamental species, including apple (R: 100, 101), pear (R: 115), plum (R: 77–79, 88, 89), cherry (R: 64, 66, 93), walnut (R: 126), aronia (R: 8), vaccinium (R: 29, 30, 34-36), pistacia (R: 127), magnolia (R: 141, 142), linden (R: 144-146), camptotheca (R:136). Some of them are propagated commercially nowadays. Application of LED lights (R: 20, 30, 88, 115), selection for drought resistance, the effect of basal medium composition on biomass accumulation and exopolysaccharides production, embryo rescue, and virus elimination (R: 77, 79, 101) are some of the successfully reported techniques applied for the clonal propagation of the Bulgarian germplasm. In addition to routine tissue culture techniques, various cell and molecular methods are used.

Croatia has made notable progress in micropropagation of various fruit tree species and in applying different methodologies (**Table 1**, **Supplementary File**, Rows-R). *In vitro* techniques have been investigated in various species such as saskatoon berry (R: 3), honeyberry (R: 10), raspberry (R: 21), blueberry (R: 31), wild cherry (R: 48), sour cherry (R: 55–57), ‘CAB-6P’, ‘Gisela 6’ and ‘Gisela 5’ rootstocks (R: 71–72), apple (R: 102–103), satsuma mandarin (R: 131), grape (R: 148–149). The research includes the application of successful sterilization techniques and media composition, the incorporation and investigation of next-generation temporary immersion bioreactor systems (TIS/TIB systems) in micropropagation, testing the effect of light—combinations of red and blue LED lamps with conventional fluorescent lamps, and inducing stress resistance under drought conditions. Thermotherapy, micrografting, meristem culture, cryotherapy, and cryopreservation trials are the most used techniques for micropropagation, sanitation, and preservation of Croatian germplasm.

Greece has directed its research efforts towards improving the propagation of economically important species for the agricultural industry, such as olive trees, grapevines, and certain fruit trees (**Table 1**, **Supplementary File**, Rows-R). Advanced clonal propagation techniques are used to maintain uniformity and improve traits of interest (disease resistance, growth rate, fruit production and quality). The research focuses mainly on improving the efficiency and cost-effectiveness of micropropagation through through optimization of nutrient nutrient media composition, sterilization protocols, and rooting techniques. Micropropagation research covers a wide range of species, including golden kiwifruit (R: 2), Greek strawberry tree (R: 5), hybrid strawberry (R: 6), strawberry tree (R: 7), cade juniper (R: 9), myrtle (R: 14), olive cultivars (R: 37–38), weeping pittosporum (R: 39), *Prunus* rootstocks [‘GF 677’ (R: 41), ‘PR 204/84’ (R: 42), ‘MxM’ (R: 50), ‘CAB-6P’ and ‘Gisela 6’ (R: 73), ‘SL 64’ (R: 74), ‘Krymsk 86’ (R: 75), ‘Krymsk^®^ 5’ (R: 92)], apple rootstocks [‘EM 9’ (R: 104), ‘MM 106’ (R: 105), ‘M 9’ (R: 106), ‘M 4’ (R: 107)], pear (R: 116), almond-leaved pear (R: 122), sour orange (R: 128), citrus hybrid (R: 129), pomelo (R: 130), citrus rootstocks (R: 132–134), trifoliate orange (R: 135), and grapevine cultivars [‘Agiorgitiko’ (R: 150), ‘Malagouzia’, ‘Xinomavro’ (R: 151), ‘Giouroukiko’, and ‘Serifiotiko’ (R: 152)]. The research primarily aims to optimize key micropropagation stages - proliferation, multiplication, and rooting - while also focusing on reducing oxidative stress and improving plant health. Special attention is given to meristem culture and thermotherapy for virus-free plant production, ensuring the sustainability and resilience of important fruit and nut crops in Greece. Finally, the possibility of introducing new species in the floriculture industry such as, *Ebenus sibthorpii* (R: 139) and *Senna artemisioides* (R: 143), by using micropropagation techniques is being investigated.

Serbia stands out for its extensive research on various fruit crops and *in vitro* methodologies (**Table 1**, **Supplementary File**, Rows-R). Key research areas include: (i) *In vitro* cloning - studies have mainly focused on optimizing conditions for establishing aseptic cultures, plant multiplication, rooting, and acclimatization of berry fruits [black currant (R:19), raspberry (R: 22–23), blackberry (R: 26), blueberry (32)], stone fruits [wild cherry (R: 47), sweet cherry (R: 49), sour cherry (R: 58–60), mahaleb cherry (R: 96), steppe cherry (R: 91), cherry rootstocks (R: 51, 61, 65, 68, 94), cherry plum (R: 52), plum (R: 80), plum rootstocks (R: 90, 97)], pome fruits [apple (R: 108, 110–111), pear rootstocks (R: 117–118)], and ornamentals [carob (R: 137), wintersweet (R: 138)]. Additionally, gross genetic fidelity in these cultures has been assessed through flow cytometry, light microscopy, and isozyme analysis (R: 25, 60, 67, 117). Performance of *in vitro* propagated plants was also compared with traditionally propagated ones in the open field, focusing on yield and fruit quality, including phenolic and volatile compounds (R: 23, 26, 84); (ii) *In vitro* preservation – slow-growth storage has been used to preserve *in vitro* collections of various temperate fruit tree species at FRI, Čačak. This method supports short- and medium-term preservation, easy exchange of plant material, and allows for rapid propagation when necessary. Studies have focused on shoots of plum (R: 81, 85), cherry plum (R: 53), sour cherry (R: 62), cherry rootstock (R: 69), apple (R: 108), raspberry (R: 24), as well as encapsulated shoot tips of berry species (R: 24, 27). A decade-long research program on cryopreservation has optimized techniques like encapsulation-dehydration (R: 28, 54) and vitrification (R: 70, 109), and their modifications [droplet vitrification (R: 28, 70, 82, 109), V cryo-plate and D cryo-plate (R: 4, 33, 54, 70, 82, 86)] for preserving fruit species. The aim was not only to improve regrowth success but also to ensure proper multiplication, rooting, and acclimatization of cryopreserved plants; (iii) Virus-free plant production – the widespread presence of plum pox virus (PPV) in Serbia led to the development of *in vitro* techniques for virus eradication. Chemotherapy with ribavirin has been used to eliminate PPV from plum cultivars bred at FRI (R: 87), while cryopreservation methods (V cryo-plate and D cryo-plate) were also tested for their efficiency in eradicating PPV from autochthonous plums (R: 83); (iv) Secondary metabolites production – the potential of *in vitro* propagation for producing secondary metabolites was explored in small fruit species. *In vitro*-grown leaves of blackberry and blueberry showed higher phenolic content and antioxidant activity compared to callus cultures, indicating their value for secondary metabolites production (R: 26); (v) Genetic transformation – *Agrobacterium rhizogenes*-mediated transformation of different apple cultivars (R: 112) has provided a new tool for investigating apple allelopathy.

**Figure 2:**
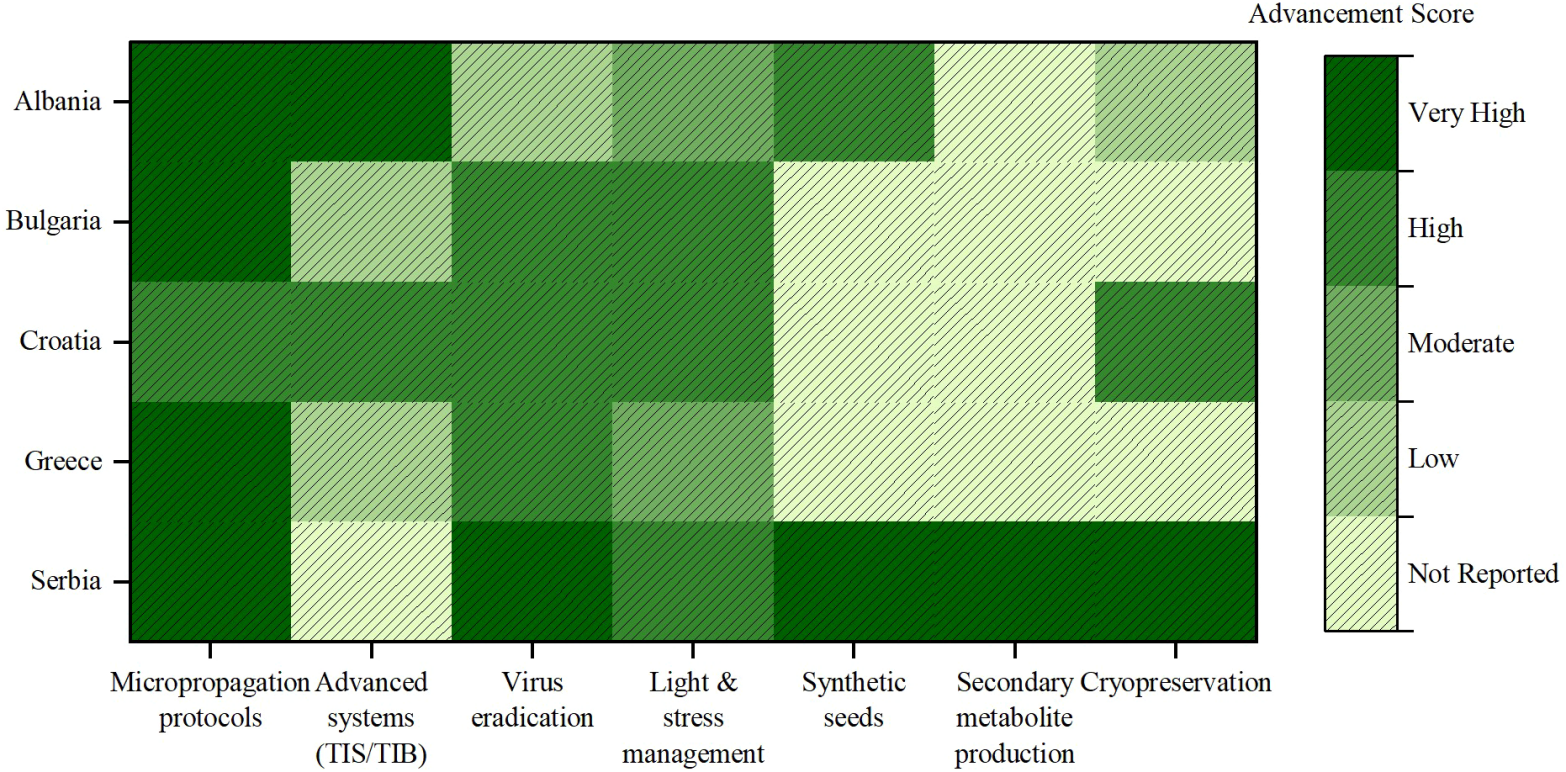
Heatmap illustrating the level of advancement in key *in vitro* biotechnology domains across Balkan countries regarding fruit trees and woody ornamental species.

According to the statistical analysis of the table content (**Table 1**, **Supplementary File**), Serbia leads with the highest number of studies (31.33%), followed by Greece (23.49%), Albania and Bulgaria (each with 18.07%), and Croatia (9.04%). As presented in **Figure 2**, Serbia shows advanced engagement in almost all areas (except the use of bioreactors in the propagation of woody plants), making the country a regional pioneer and a potential center for capacity building. Albania has advanced capabilities in synthetic seed production and micropropagation in bioreactors. Trials on long-term storage through cryopreservation have only just begun, while the production of secondary metabolites has not yet been researched. Bulgaria has made considerable progress in the application of light and stress physiology, as well as micropropagation of commercially relevant species; however, it has comparatively less experience in long-term conservation technologies and TIS systems. Greece, which specializes in the propagation of elite varieties and the eradication of viruses, has extensive expertise in producing virus-free plants and standardizing plant material for agricultural use. Croatia is positioning itself as a science-based player with clear potential for further progress in this area. It demonstrates a significant commitment to developing micropropagation protocols, utilizing temporary immersion bioreactors, implementing virus eradication strategies, managing light and stress conditions, and introducing cryopreservation. The heatmap highlights the importance of knowledge transfer and alignment of specialization through the following priorities: (i) the extension of micropropagation and *in vitro* conservation protocols to underrepresented ornamentals and indigenous fruit tree species, which represent a valuable genetic resource for the selection of clones resistant to economically important diseases and for the breeding of new varieties; (ii) the development of scalable, stress-resistant systems for difficult-to-reproduce woody fruit tree genotypes; and (iii) the strengthening of infrastructure and knowledge transfer in countries where these tools are being developed.

#### 2.2.2 Forest plant species

Only three countries in the Balkans have been working on applying *in vitro* techniques for forest plant species in the last two decades—Bulgaria, Greece, and Serbia (**Table 2**; **Supplementary File**, Rows-R).

Bulgaria’s scientific research was mainly focused on optimizing the micropropagation stages for various species, such as *Fraxinus* sp. (R: 11), *Acer* sp. (R: 1), *Ginkgo* sp. (R:12), *Betula* sp. (R: 9), *Ilex* sp. (R: 13), *Paulownia* sp. (R: 15), *Populus* sp. (R: 20, 21, 24, 26, 27), *Quercus* sp. (R: 28, 29), and *Sorbus* sp. (R:33). Some of the scientific articles include vitrification for long-term storage (R: 21), adventitious shoot regeneration (R: 9, 11, 20), encapsulation of microcuttings (R: 25, 27, 29, 30, 34), etc.

Greece is seeing increasing interest in forest species such as *Ilex aquifolium* L (R: 13), an ornamental species mainly used for decoration during Christmas. The research focuses mainly on the development of vegetative propagation protocol and the increase of rooting capacity. Many studies aim to develop efficient methods for propagating endangered or threatened native Greek woody plant species such as *Quercus euboica* Pap. (R: 30), a rare, endangered oak species thatcan be used as an ornamental plant and for the reforestation of urban and suburban areas.

Serbia’s studies on *in vitro* cloning include trees, ornamental and medicinal shrubs, and endemic conifers. All investigations in this century include: (i) Different ways of propagation: androgenesis, somatic embryogenesis, and organogenesis. Two types of cultures were used to induce androgenesis in *Aesculus* species: anther and microspore suspension culture. Somatic embryogenesis was achieved in the culture of stamen filaments, and *de novo* shoot bud induction was achieved from somatic seedlings (R: 2–8). The protocols for micropropagation have been developed for *Paulownia elongata* (R: 14). Only in Serbia, there is certain progress on conifer cloning of the Tertiary relict species endemic to the Balkan Peninsula, like Serbian spruce, Bosnian and Macedonian pine. Somatic embryogenesis and adventitious bud formation of Serbian spruce were induced in parallel on a cytokinin containing medium. Micropropagation of Bosnian pine was achieved in cultures of mature zygotic embryos,and somatic embryogenesis was induced in cultures of isolated megagametophytes. Immature cleavage polyembryos were shown to be at the most suitable stage for the induction of embryogenic tissue in Bosnian pine. Micropropagation of Macedonian pine has been achieved in zygotic embryo culture and juvenile plant material using a short-term liquid cytokinin pulse (R: 16–19); (ii) Production of valuable secondary metabolites during *in vitro* propagation and after genetic transformations was evaluated for *A. hippocastanum* and *Rhamnus fallax* Boiss. The highest aescin content was detected in horse chestnut androgenic embryos at the cotyledonary stage (R: 2). Hairy roots of *A. hippocastanum* obtained after genetic transformation produced aescin in lower concentration compared to zygotic embryos which might be compensated by the high biomass production of hairy roots (R: 2). Hairy roots of *R. fallax* showed an increase in anthraquinone content (R: 31, 32); (iii) *In vitro* selection of tolerant genotypes for abiotic stress tolerance (drought, salinity, acidity and elevated heavy metal concentrations). These investigations included the model tree species (*Populus alba* and *P. nigra*). White poplar (*P. alba*) is an endangered species in Serbia where the tissue culture represents an important propagation technique (R: 22). Poplars are used for phytoremediation and potentially large quantities of heavy metals could be extracted such as lead and nickel (R: 23). Copper accumulation in *P. nigra* strongly depends on the genotype (R: 25).

**Figure 3:**
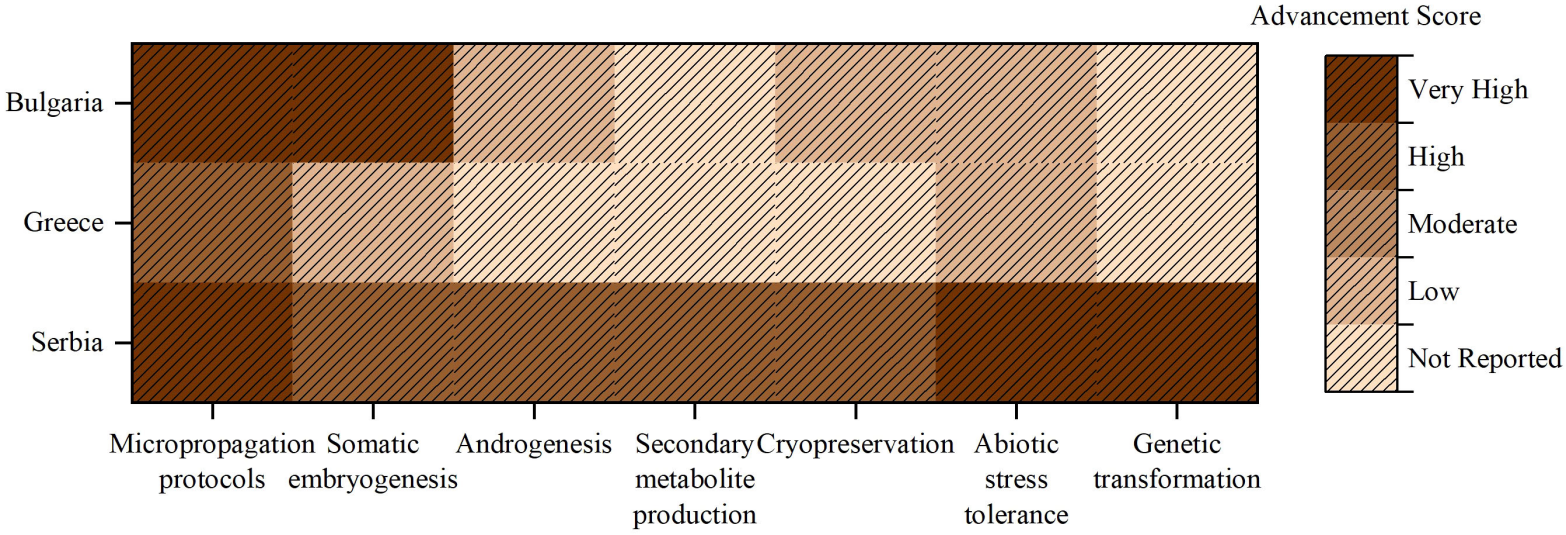
Heatmap illustrating the level of advancement in key *in vitro* biotechnology domains across Balkan countries regarding forest tree species.

According to the statistical analysis of the table content (**Table 2**, **Supplementary File**), Serbia has the highest number of studies (58.33%), followed by Bulgaria (36.11% each) and Greece (5.56%). Over the last decade, the use of *in vitro* techniques in forest species in the Balkans has been very uneven, showing both a leading role and gaps between countries (**Figure 3**). Serbia stands out as a regional pioneer, having introduced a wide range of advanced methods such as micropropagation, somatic embryogenesis, Androgenesis, and genetic transformation. Bulgaria has demonstrated strong capacities in adventitious shoot regeneration, encapsulation techniques, and cryopreservation, especially in *Populus* and *Quercus* species. However, its commitment to more complex biotechnological applications remains limited. While Greece is growing interested in rare ornamental forest species, it has yet to move beyond conventional micropropagation. This analysis highlights the need for targeted investment and capacity building in under-represented techniques such as somatic embryogenesis and stress selection in Bulgaria and Greece. Coordinated regional initiatives, knowledge transfer, and infrastructure support could accelerate the harmonization of forest biotechnology and promote the sustainable conservation of genetic resources in the Balkans.

#### 2.2.3 Emerging commercial micropropagation of woody plants in the Balkans

Over the past few decades, commercial *in vitro* plant cultivation has experienced significant growth throughout Europe. Every year, between 70 and 100 million microplants are produced in about 140 commercial *in vitro* labs across Europe (Podwyszyńska et al., 2022). Although numerous countries have made considerable investments in state-of-the-art tissue culture facilities designed for large-scale plant multiplication, the commercial application of *in vitro* plant biotechnology in the Balkans is still at an early stage. Many of these commercial labs are locally owned, though others are branches of Western European companies, probably due to the region`s lower operational costs. A distribution map of commercial labs (**Figure 2**) provides further insight into the concentration and expansion of these facilities throughout the region.

Bulgaria’s advancements in agricultural biotechnology are exemplified by the efforts of private enterprises and academic research institutions. At the moment, two private labs—one owned by Industrial Plants Ltd. in Kazanlak (https://industrial-plants.net) and another connected to the Institute of Fruit Growing in Plovdiv (https://fruitgrowinginstitute.com)—are involved in the large-scale micropropagation of a variety of species, including kiwis, blueberries, raspberries, some medicinal plants, and fruit rootstocks.

In Croatia, commercial tissue culture remains relatively small, with only two active labs: PhytoCulture d.o.o., Donje Podotočje (Data provided by HAPIH – Croatian Agency for Agriculture and Food, Centre for Seed and Seedlings, www.hapih.hr) and Biotech - Regional Center for Biotechnology Research and Development, Slavonski Brod (https://www.biotech.hr). These labs specialize in large-scale production of fruit, ornamental, and aquatic plants. Additionally, a new plant tissue culture facility established in 2023 at Ilok High School (https://ssilok.hr) as part of an EU-backed project will serve as a training hub for future specialists in micropropagation.

Greece has seen considerable advancement in large-scale micropropagation, particularly for woody species. VITRO HELLAS was founded in 1986 in Niseli Alexandria, Imathia, Northern Greece (https://www.vitrohellas.gr/en/home), and a tissue culture lab was officially opened the following year. The company is involved in developing, producing, and distributing high-quality plant propagation materials. It collaborates with both Greek and international universities and research institutes, producing planting material for a wide range of fruit tree species such as apples, cherries, apricots, peaches, pears, almonds, kiwi, plums, as well as rootstocks for apple, cherry, peach, nectarine, apricot, and plum trees. Newer enterprises, such as Vitrogreen L.P., founded in 2017 (https://www.vitrogreen.gr), and FITOTECHNIKI, founded in 2019 (https://fitotechniki.com), are located in Filothei-Arta and specialize on kiwi and rootstocks production. The growing interest from nurseries shows that Greece’s *in vitro* sector will continue to expand.

North Macedonia’s commercial micropropagation sector is also expanding, with two companies specializing in woody plants and one focused on large-scale production of herbaceous ornamentals. A notable aspect of this country’s sector is that two companies are branches of European enterprises operating in the sector, since such investment is considered cost-effective due to low production costs. SBW Romero Vitro, established in 2001 in Vinica (https://iribov.com/about/#macedonia) as a branch of the Dutch company Iribov, propagates over 5000 varieties of ornamental and berry plants. Plant Engineering, founded in 2018 in Skopje (https://www.instagram.com/plantengineering/), focuses on *in vitro* production of various vegetable crops, fruit, medicinal and aromatic plants. Additionally, Anthura MK (https://anthura-mk.com/en), a branch of Anthura BV from the Netherlands located in Kochani, specializes in the micropropagation of ornamental plants such as Anthurium, Bromeliad, and Phalaenopsis.

Serbia began commercial micropropagation of woody plants 15 years ago by establishing the first commercial lab at Superior d.o.o. in Velika Plana (https://superior-seeds.co.rs). The company focuses on the propagation of various fruit tree cultivars and rootstocks. More recently, InVitroS - established in 2020 in Budisava (https://invitros.rs), and Florand nursery - established in Subotica (https://www.paulovnijadrvo.rs), have expanded production, focusing on flowers, berries, fruit rootstocks, and species like *Paulownia*. The Fruit Research Institute (https://institut-cacak.org) in Čačak also contributes to the market by producing *in vitro* basic and certified planting material of berry fruits and autochthonous plums. The basic planting material of raspberry, obtained by micropropagation, is used to establish nurseries that generate certified planting material for commercial sale.

Meanwhile, Albania, Bosnia and Herzegovina, Kosovo, and Montenegro still lack adequate commercial micropropagation systems.

**Figure 4:**
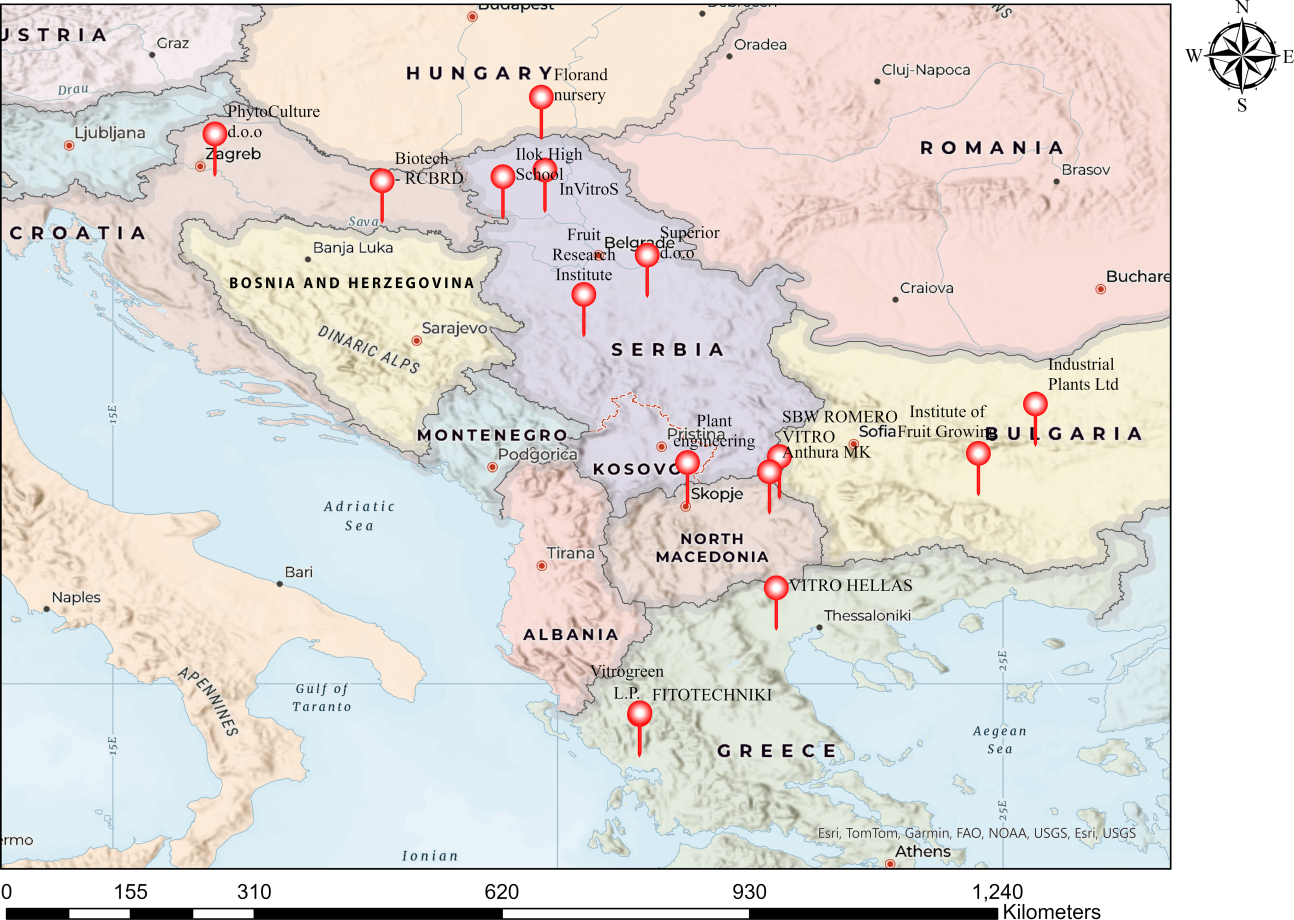
Distribution of commercial micropropagation labs in the Balkans (Source: companies’ websites).

It can be concluded that commercial *in vitro* laboratories are increasingly common in the Balkans, particularly in countries such as Bulgaria, Croatia, Greece, Serbia and North Macedonia (**Figure 4**); however, their integration into broader agricultural and scientific applications remains uneven. Collaboration between private laboratories and public research organizations is still limited. Notable exceptions include joint initiatives in Bulgaria and scientific alignment of scientific objectives between commercial laboratories and academic institutions in Greece. Despite these efforts, structured public-private partnerships remain underdeveloped, limiting technology transfer, protocol optimization, and the market introduction of new plant varieties. The expansion of commercial *in vitro* laboratories in the region is constrained due to several obstacles, including the lack of qualified personnel with specialized tissue culture training, the absence of standardized certification systems for *in vitro*-derived plant material, and insufficient policy measures at the national level.

On the other hand, inconsistent legislation in the Balkan countries makes it difficult for companies to comply with EU-wide quality and phytosanitary standards. Financial challenges are another major obstacle. The high initial investment required to set up and operate modern, high-tech tissue culture facilities is often prohibitively expensive, posing a particular challenge in countries with underdeveloped markets. Most companies rely on limited, inadequate local funding sources to achieve full commercialization, although some benefit from EU-funded initiatives or foreign direct investment, such as the Dutch-backed initiatives in North Macedonia. Despite these obstacles, the European market for plant tissue culture offers significant opportunities. The European plant tissue culture market is expected to grow at a CAGR of 8.6%, from $128.52 million in 2022 to $211.16 million in 2028 (Europe PTC Market, 2022). The Balkan countries, where growing investments, collaborations, and technological developments are making the region a major center for *in vitro* plant biotechnology, will benefit significantly from this expanding sector. The map of commercial laboratory distribution (**Figure 4**) reflects existing activities and highlights strategic hotspots for future investments, emphasizing the need for coordinated regional development strategies.

## 3 Regional progress, constraints, and future perspectives

**Figure 5:**
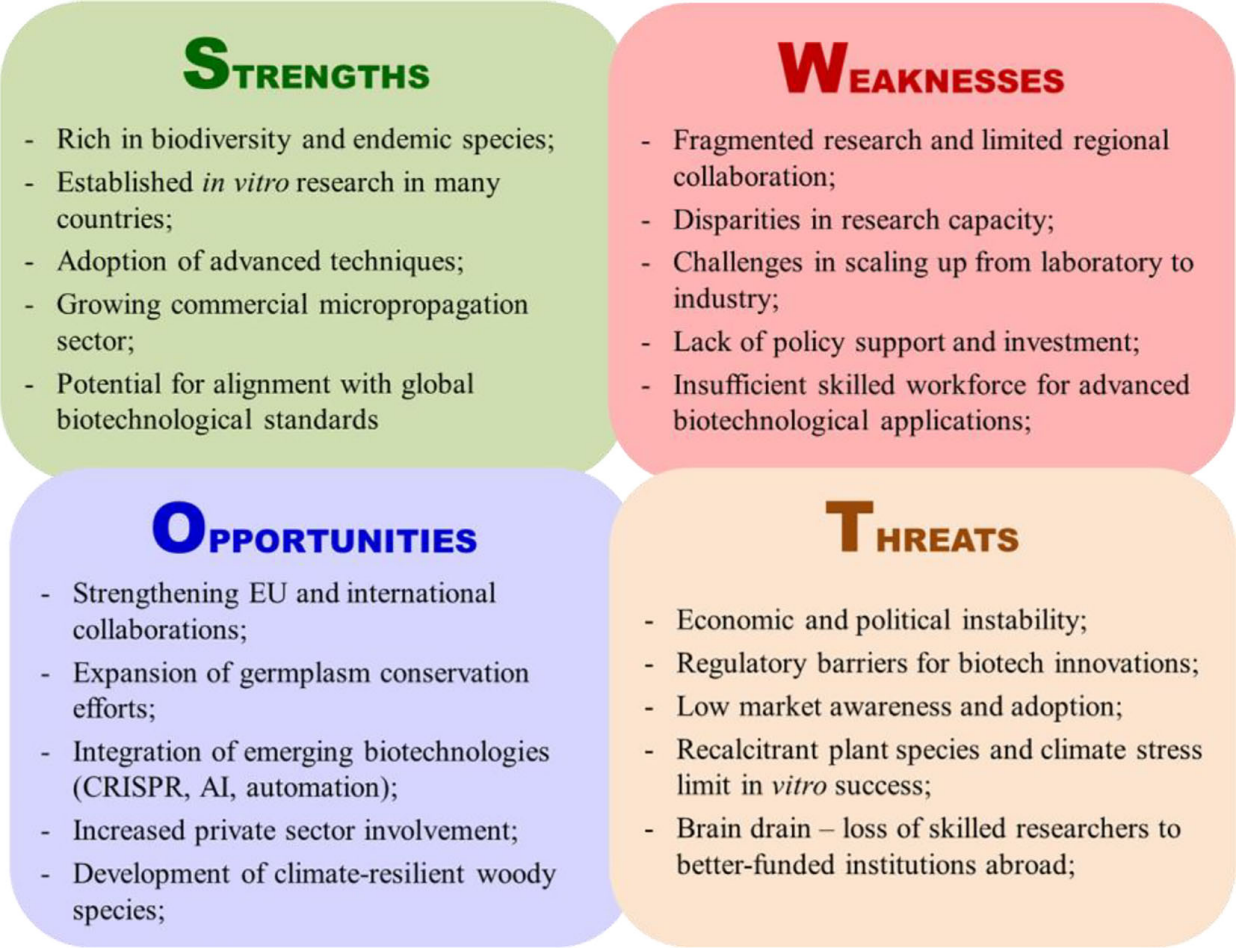
SWOT analysis on *in vitro* woody plants biotechnology in the Balkans.

This analysis offers the first thorough review of *in vitro* biotechnological applications in woody plants throughout the Balkan Peninsula. It helps identify key achievements, gaps, and constraints that limit large-scale implementation and commercialization (**Figure 5**). This makes informed decision making possible for different stakeholders when creating focused plans for policy alignment, industry-academia collaborations, and scientific integration.

### 3.1 Key findings: strengths and weaknesses

#### 3.1.1 Strengths

The region has a strong biotechnological capacity in the field of plant tissue culture and propagation. Significant progress has been made in the selection of stress-tolerant genotypes, *in vitro* conservation, and micropropagation, yielding results that meet global biotechnological standards. Serbia is a leader in woody plant cloning, cryopreservation, virus eradication, and large-scale plant production. Bulgaria has developed molecular-based approaches to problem-solving, embryo rescue, virus elimination protocols, and commercial micropropagation. In Greece, research has focused on the large-scale micropropagation of commercially important crops, including olives, vines, and rootstocks. Croatia has invested in thermotherapy, next-generation bioreactors, and cryopreservation trials and has also focused on commercialization. Albania has developed *in vitro* preservation techniques, synthetic seed technology, and TIS bioreactors. Bosnia and Herzegovina and North Macedonia have a well-developed infrastructure, although these countries are more focused on herbaceous plants. Serbia, Bulgaria, and, to a lesser extent, Greece have also made significant advances in scientific research on forest plant species.

#### 3.1.2 Weaknesses

*Underrepresented countries for applications in woody plant cloning:* Despite their capacity for *in vitro* research, Bosnia and Herzegovina and North Macedonia primarily focus on herbaceous and vegetable crops rather than woody plants. In North Macedonia, due to lower operating costs, commercial micropropagation companies have emerged, but instead of promoting local scientific research, several institutions operate as subsidiaries of European companies. Kosovo and Montenegro could develop more *in vitro* research facilities and scientific infrastructure.

*Limited research on forest* sp*ecies:* Despite their ecological importance, this category has received less attention than fruit and ornamental trees, resulting in a significant gap in protocols for the propagation, conservation, and restoration of forest species.

*Fragmented research efforts and limited collaboration:* Although research groups in Balkan countries have achieved notable successes, research remains primarily confined within national boundaries. A limitation on cross-border collaboration and a lack of shared databases have been observed, which slow down the regional integration of knowledge and methodologies.

*Commercialization and industry expansion constraints:* The transition from laboratory-scale micropropagation to large-scale commercial production remains limited in many countries. The use of *in vitro* techniques in commercial nurseries and forestry projects is progressing slowly due to funding constraints, a shortage of skilled workers, and a lack of solid industry-academia collaborations. The presence of foreign-owned facilities highlights the potential cost-effectiveness of production in the region, yet their impact on domestic biotechnology progress remains limited. However, many Balkan countries are not EU members, which may affect investment opportunities and policy alignment.

*Insufficient skilled workforce for advanced biotechnological applications:* Many universities in the Balkans lack specialized training programs for young researchers and professionals with the necessary practical skills. Limited funding opportunities and lower salaries compared to Western Europe result in disincentives for retaining talent in the region.

### 3.2 Future outlook: opportunities and threats for advancing regional innovation

**Figure 6:**
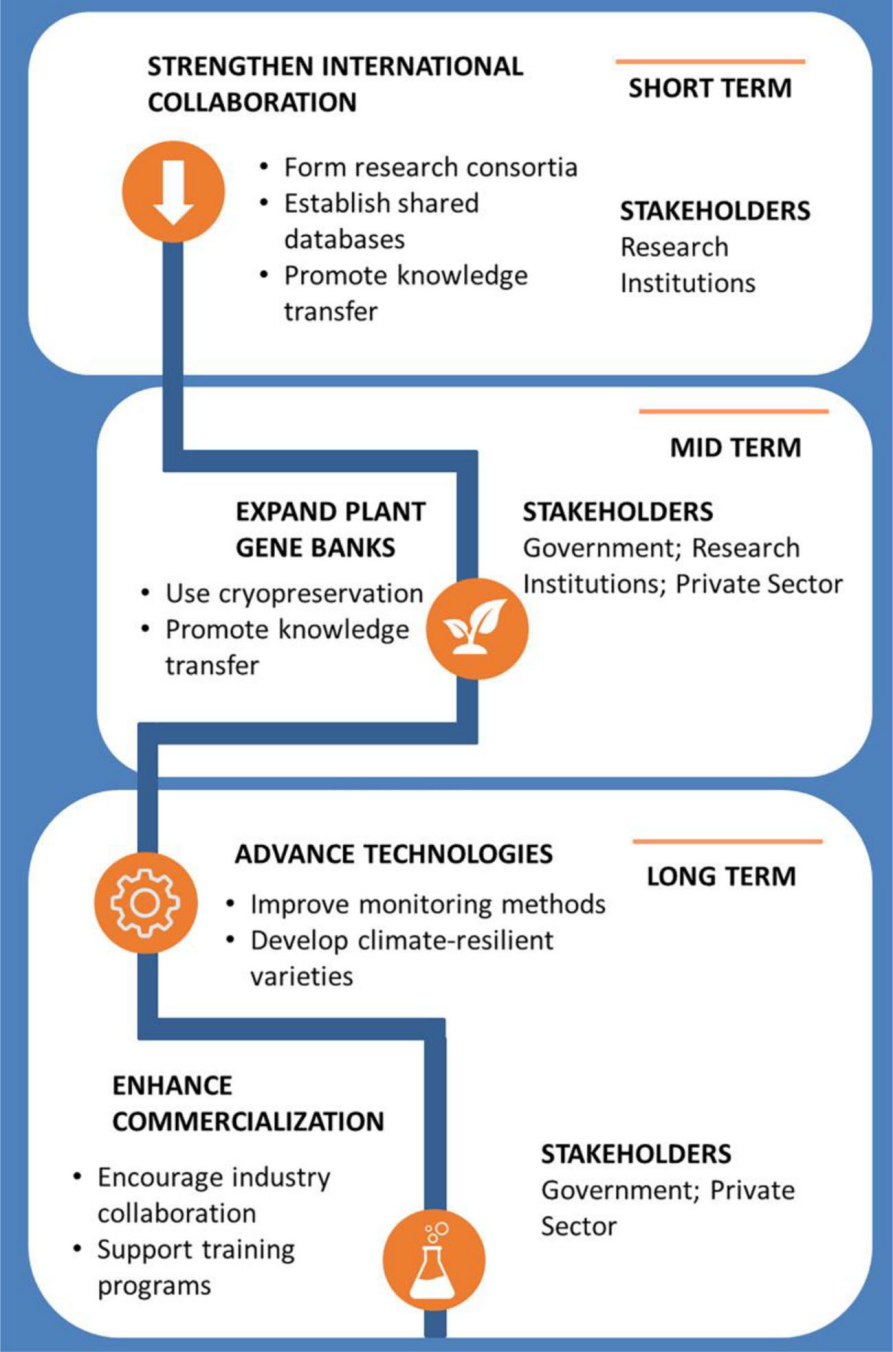
Roadmap for advancing *in vitro* research on woody plant species in the Balkans.

Although progress is being made in the Balkans in developing biotechnological methods for woody plants, the next phase of development will depend on how effectively these opportunities are utilized and how the threats are managed. As outlined, regional differences in capacity, infrastructure, and policy remain. The proposed roadmap (**Figure 6**) provides a strategic framework for advancing *in vitro* research on woody plant species. It summarises the main priorities discussed below and divides them into opportunities for further innovation and threats that could hinder progress. Each of the four main areas of development—international collaboration, genebank expansion, technological innovation, and commercialization—is also summarized visually in the diagram to aid coordination between stakeholders.

#### 3.2.1 Opportunities

Strengthening collaboration at the international level: Forming multi-country research consortia to improve funding success in European programs (Horizon Europe, COST Actions, Erasmus+) can create a more integrated approach by reducing fragmented scientific research and favoring knowledge sharing among researchers. Establishing shared databases can also facilitate knowledge sharing, especially with countries in the early stages of scientific application in this field.

*Plant gene bank expansion for long-term storage*: Using cryopreservation and synthetic seed technologies is essential to improving the long-term preservation of plant genetic resources (particularly autochthonous and endangered) in the Balkans. This approach will help address the region’s vulnerability to climate change and genetic erosion. Furthermore, promoting knowledge transfer, technical training, and capacity building through workshops, courses, and the exchange of best practices will strengthen regional expertise and collaboration. Finally, it will contribute to the sustainability of plant genetic resources conservation in Europe (Maggioni et al., 2017).

*Technological advancements*: Improving monitoring and control methods by using advanced automated sensor systems may optimize large-scale cloning conditions (Vidal and Sánchez, 2019). Selecting climate-resilient species or genotypes with inherent resistance to climate-induced stresses (Vuksanović et al., 2019) and optimizing tissue culture media by testing new compounds will be essential for long-term success (Wijerathna-Yapa and Hiti-Bandaralage, 2023). Biotechnological innovations such as genetic engineering or gene editing (e.g., CRISPR technology) could help create climate-resilient plant varieties better suited to *in vitro* propagation and post-propagation conditions (Cardi et al., 2023).

*Commercialization and private sector involvement*: If governments and investors encourage innovation, more biotech and startup businesses, like those currently operating in the Balkan countries, may arise. Research could be applied commercially by closer cooperation with forestry and nursery businesses if they were more confident of the potential benefits. This would also support training programs and scientific visits for researchers and students in the Balkans, increasing human capacity for advanced scientific research.

#### 3.2.2 Threats

*Economic and Political Instability*: Some Balkan countries face economic constraints and political instability, which reduces government focus and funding on agricultural biotechnology. The EU accession process is slow, delaying access to funding and harmonized policies. On the other hand, this is also associated with limited market awareness. Farmers and commercial growers may be unaware of the benefits of *in vitro*-derived plants, leading to low adoption rates. Furthermore, due to economic and social reasons, the rate of brain drain is increasing in many Balkan countries, particularly in the Western Balkans (Vracic, 2018). Limited career opportunities, lower salaries, and insufficient investment in scientific infrastructure drive many skilled researchers to seek opportunities abroad.

*Climate-related stress limits in vitro techniques*: Slowing plant growth can reduce shoot multiplication and rooting (Vuksanović et al., 2020). Alterations in nutrient uptake and metabolism of mother plants complicate the development of standardized media for *in vitro* propagation under changing environmental conditions (Kovačević et al., 2020). Climate change may also impact plant genetic diversity in natural populations, the response and adaptation of genotypes to *in vitro* conditions (Hazarika et al., 2006).

*Plant recalcitrance*: The complex biology of woody means each species and some cultivars within the same species may exhibit unique biological characteristics (Galović et al., 2014). Despite the efforts of research groups to optimize micropropagation, the results show that many forest and woody fruit species remain recalcitrant to *in vitro* culture, requiring particular conditions for optimal plant growth and development.

Moving forward and strengthening multidisciplinary regional cooperation is necessary to ensure the sustainable development of *in vitro* plant biotechnology in the Balkans. Investment in innovative biotechnological approaches and integrating scientific research with commercial applications will enable higher environmental sustainability and economic growth. This first cooperation between the Balkan countries to recognize the current state of scientific research in plant biotechnology in the Balkans is an example of a step forward in this direction. It will help ensure long-term sustainability in plant conservation and agricultural innovation.

## Footnotes

1 This designation is without prejudice to positions on status, and is in line with UNSCR 1244/1999 and the ICJ Opinion on the Kosovo declaration of independence.

## References

[B1] Allcock J. B. Crampton R. J. Danforth L. (2024). 'Balkans'. Encyclopedia Britannica. Available at: https://www.britannica.com/place/Balkans (Accessed July 1, 2025).

[B2] Atanassov A. (1995). Bulgarian plant biotechnology. Biotechnol. Biotechnol. Equip. 9, 23–37. doi: 10.1080/13102818.1995.10818849

[B3] Atanassov A. Batchvarova R. (2002). Challenges in front of the Bulgarian plant biotechnology. Biotechnol. Biotechnol. Equip. 16, 21–25. doi: 10.1080/13102818.2002.10819178

[B4] BIOFOR (2003). Biodiversity conservation and sustainable forestry. Eds. Dave C. Gibson R. S. Samir D. (Bosnia and Herzegovina. Bosnia and Herzegovina Biodiversity Assessment, prepared for USAID/Bosnia and Herzegovina, by Chemonics International, Inc). Available online at: http://pdf.dec.org/pdf_docs/PNACW775.pdf (Accessed July 1, 2025).

[B5] Cardi T. Murovec J. Bakhsh A. Boniecka J. Bruegmann T. Bull S. E.. (2023). CRISPR/Cas-mediated plant genome editing: outstanding challenges a decade after implementation. Trends Plant Sci. 10), 1144–1165. doi: 10.1016/j.tplants.2023.05.012, PMID: 37331842

[B6] Convention on Biological Diversity (2022). CBD: *Country Profiles—North Macedonia, Sixth National Report to the United Nations Convention on Biological Diversity* (Skopje, Macedonia: CBD). Available online at: https://www.cbd.int/doc/nr/nr-06/mk-nr-06-en.pdf.

[B7] Dimopoulos P. Raus Th. Bergmeier E. Constantinidis Th. Iatrou G.. (2016). Vascular plants of Greece: An annotated checklist. Supplement. Willdenowia 46, 301–347. doi: 10.3372/wi.46.46303

[B8] EEA (2023). European Environment Agency: The European reporting framework for adaptation. Available online at: https://www.eea.europa.eu/publications/is-europe-on-track-towards-climate-resilience (Accessed 17 February 2025).

[B9] Europe PTC Market (2022). Europe plant tissue culture market. Historic data: 2020-2021; forecast period: 2023-2028. Available online at: https://www.businessmarketinsights.com/reports/europe-plant-tissue-culture-market (Accessed 17 February 2025).

[B10] Galović V. Pilipović A. Marković M. Vasić V. Pap P. Pekeč S.. (2014). Nove biotehnologije u šumarstvu Srbije. Glasnik šumarskog fakulteta (Specialno izdanje), 141–156. doi: 10.2298/GSF14S1141G

[B11] Giannini R. von Wühlisch G. (2009). Workshop COST E52 “Evaluation of beech genetic resources for sustainable forestry. iForest 2, 104. Available at: http://www.sisef.it/iforest/show.php?id=500 (Accessed July 1, 2025).

[B12] Hansjörg H. Seefeld-Hechendorf B. (2022). Biodiversity and nature conservation in the Western Balkans – civil society, (local) politics, international actors, and the media in dialogue. Südosteuropa Mitt. pp, 132–140.

[B13] Hazarika B. N. Teixeira da Silva J. A. Talukdar A. (2006). “Effective acclimatization of in vitro cultured plants: methods, physiology and genetics,” in ., Floriculture, Ornamental and Plant Biotechnology: Advances and Topical Issues, Volume II. Ed. Teixeira da Silva J. A. (London, Global Science Books), 427–438.

[B14] Kongjika E. Zeka Zh. Çaushi E. Dinga L. (1995). Metodat e bioteknologjise "*in vitro*" per fitimin e bimeve te painfektuara. Punime te Institutit të Kërkimeve Biologjike 10, 37–43.

[B15] Kovačević B. Tišma G. Nikolić N. Vuksanović V. Orlović S. (2020). *In vitro* lead tolerance testing in white poplar genotypes on acidic medium. South-East Eur. Forestry 11, 153–160. doi: 10.15177/seefor.20-18

[B16] Lovrić M. Lovrić N. (2013). Integration of nature protection in forest policy in Croatia. INTEGRATE Country Report for Croatia. European Forest Institute, EFICEEC – EFISEE Regional Office. Available online at: https://efi.int/sites/default/files/files/publication-bank/projects/Croatia.pdf (Accessed 17 February 2025).

[B17] Maggioni L. López Noriega I. Lapeña I. Holubec V. Engels J. M. M. (2017). “Collecting plant genetic resources in Europe: A survey of legal requirements and practical experiences,” in Implementing the nagoya protocol (Leiden, Netherlands: Brill), 327–362. doi: 10.1163/9789004293212_016

[B18] Matevski V. (2013). Raznovidnost i poteklo na florata na republika makedonija. In: Opening adresses, contributions and bibliography of the new members of the Macedonian Academy of sciences and Arts. 17, 125–186 (Skopje, North Macedonia: Makedonska Akademija na naukite i umetnostite).

[B19] Millaku F. A. Rexhepi F. E. Krasniqi E. L. Pajazitaj Q. A. Mala X. H. Berisha N. A. (2013). The Red Book of vascular flora of the Republic of Kosovo (Prishtina: Ministry of Environment and Spatial Planning (MESP).

[B20] Ministry of Spatial Planning and Environment (2010). Fourth national report of Montenegro to the convention on biological diversity. Eds. Bušković V. Kapa M. (Podgorica: Ministry of Spatial Planning and Environment).

[B21] Müller D. Hofmann M. (2022). Impacts of climate change on agriculture and recommendations for adaptation measures in the Western Balkans. (GFA Consulting Group on behalf of the Federal Ministry of Food and Agriculture (BMEL). Leibniz Institute of Agricultural Development in Transition Economies (IAMO).

[B22] Panjković B. Rat M. Mihajlović S. Galambos L. Kiš A. Puzović S.. (2021). “Invasive alien species in the Balkan Peninsula,” in Invasive Alien Species: Observations and Issues from Around the World. Eds. Ielmini M. R. Pullaiah T. (John Wiley & Sons, Hoboken, NJ), 42–87.

[B23] Podwyszyńska M. Orlikowska T. Trojak-Goluch A. Wojtania A. (2022). Application and Improvement of *in vitro* culture systems for commercial production of ornamental, fruit, and industrial plants in Poland. Acta Societatis Botanicorum Poloniae 91, 914. doi: 10.5586/asbp.914

[B24] Polunin O. (1980). Flowers of Greece and the balkans-A field guide (Oxford: Oxford University Press).

[B25] Radojević Lj. Vujičić R. Nešković M. (1975). Embryogenesis in tissue culture of *Corylus avellana* L. Z. Phlanzen Physiol. 77, 33–41. doi: 10.1016/S0044-328X(75)80123-1

[B26] Reed J. M. Kryštufek B. Eastwood W. J. (2004). “The physical geography of the Balkans and nomenclature of place names,” in Balkan Biodiversity: Pattern and Process in the European Hotspot, vol. pp . Eds. Griffiths H. I. Kryštufek B. Reed J. M. (Springer, Dordrecht), 9–22. doi: 10.1007/978-1-4020-2854-0_2

[B27] Šijačić-Nikolić M. Milovanović J. Nonić M. (2014). Forest genetic resources in Serbia - state and recommendations for improvement in this area. Bull. Faculty Forestry, 51–70. doi: 10.2298/GSF14S1051S

[B28] Stoyanov K. Raycheva T. Cheschmedzhiev I. (2022). “Key to the native and foreign vascular plants in Bulgaria,” in Interactive extended and supplemented edition (Academic Publishing House of the Agrarian University, Plovdiv). doi: 10.13140/RG.2.2.36390.95047

[B29] Turill W. B. (1929). The Plant-Life of the Balkan Peninsula - a Phytogeographical Study (Oxford: Clarendon Press).

[B30] Vidal N. Sánchez C. (2019). Use of bioreactor systems in the propagation of forest trees. Eng. Life Sci. 19, 896–915. doi: 10.1002/elsc.201900041, PMID: 32624981 PMC6999064

[B31] Vracic A. (2018). The way back: brain drain and prosperity in the Western Balkans (European Council on Foreign Relations). Available online at: http://www.jstor.org/stable/resrep21635 (Accessed February 25, 2025).

[B32] Vuksanović V. Kovačević B. Kesić L. Pavlović L. Vaštag E. Kebert M. (2020). Effect of IBA and TIBA on rhizogenesis of Wild cherry *in vitro* . Topola 206, 5–11. doi: 10.5937/topola2006005

[B33] Vuksanović V. Kovačević B. Orlović S. Kebert M. Kovač M. (2019). The influence of drought on growth and development of white poplar shoots in *vitro* . Topola/Poplar, 13–18.

[B34] Wijerathna-Yapa A. Hiti-Bandaralage J. (2023). Tissue culture—a sustainable approach to explore plant stresses. Life 13, 780. doi: 10.3390/life13030780, PMID: 36983935 PMC10057563

[B35] Yordanov M. (1986). Cutting-edge technologies in the agriculture of Plovdiv district (Sofia, Bulgaria: Zemizdat).

[B36] Zeneli G. Shuka L. Bego F. Kashta L. Shumka S. Hoxha S.. (2014). National biodiversity strategy of Albania, (2012-2020) (Tirana: Ministry of Environment). doi: 10.13140/RG.2.2.31927.93606

