# Supplementary material for: Unveiling the Balkans’ advances: In vitro biotechnology of woody plants in the early 21st century
Source: Front Plant Sci. 2025 Aug 25;16:1586013. doi: 10.3389/fpls.2025.1586013 (PMC12415014; doi:10.3389/fpls.2025.1586013)
Supplement: Supplementary file 1 [file DataSheet1.pdf]

Supplementary File

TABLE 1 Scientific research on *in vitro* plant tissue culture of woody crops and ornamentals in the Balkan countries (2001–2024)

Fruit species	Common name /Cultivar/Genotype	Aim of the research	Reference	*Institutions/Country	Row No. (R)
**Berry fruits**					
Actinidia deliciosa (A. Chev.) CF Liang et AR Ferguson	kiwifruit	Microprogation using stem pieces	Kongjika et al. (2003)	ASA, AL	1
Actinidia chinensis Planch.	golden kiwifruit	*In vitro* regeneration	Sotiropoulos and Dimassi (2004)	ELGO-DEMETRA GR	2
Amelanchier alnifolia (Nutt.)	saskatoon berry	Multiplication; chlorophyll content; stomatal formation	Bošnjak Mihovilović et al. (2020)	UNiZG – FAZ, HR	3
		Cryopreservation (V and D cryo-plate)	Vujović et al. (2024a)	FRI, RS	4
Arbutus andrachne L.	greek strawberry tree	Micropropagation	Bertsouklis and Papafotiou (2009); Papafotiou (2013a)	AUA, GR	5
Arbutus × *andrachnoides* Link	hybrid strawberry	Micropropagation	Bertsouklis and Papafotiou (2011); Papafotiou (2013a; 2013b)	AUA, GR	6
Arbutus unedo L.	strawberry	Micropropagation	Mereti et al. (2002; 2003); Papafotiou (2013a)	Vitro Hellas S.A., GR; AUA, GR	7
Aronia melanocarpa (Michx) Elliott	black chokeberry	Suspension culture	Krasteva et al. (2023)	IM-BAS, BG	8
Juniperus oxycedrus L	cade juniper	*In vitro* regeneration	Bertsouklis et al. (2023a)	AUA, GR	9

Lonicera caerulea L.	haskap berries ‘Kalinka’, ‘Polar Jewell’, ‘Balalaika’	Establishment, multiplication and rooting	Mihaljević et al. (2019)	PIO, HR	10
Myrtus communis L.	myrtle	Micropropagation; Slow growth conservation	Sota and Kongjika (2010); Kongjika and Sota (2018)	Biotech-UT, AL; ASA, AL	11
		Glandular trichomes density; *In vitro* propagation	Sota et al. (2020); Kongjika and Sota (2018)	Biotech-UT, AL; ASA, AL	12
		ElecTIS bioreactor	Papakosta et al. (2022)	ATTC, AL	13
		In vitro rooting; Acclimatization	Hatzilazarou et al. (2003)	AUTh, GR	14
Punica granatum L	pomegranate ‘Devedishe’	Genetic diversity; Micropropagation	Bacu et al. (2009; 2013)	Biotech-UT, AL	15
		Micropropagation	Kongjika and Sota (2018); Sota et al. (2022)	Biotech-UT, AL; ASA, AL	16
		Explants’ sterilization methods	Lala et al. (2022)	Biotech-UT, AL	17
		Synthetic seeds production; Encapsulation - vitrification	Lala et al. (2024a; 2024b)	Biotech-UT, AL	18
Ribes nigrum L.	black currant ‘Čačanska Crna’	Micropropagation (PGRs; explant orientation)	Ružić and Lazić (2006); Vujović et al. (2012c)	FRI, RS	19
Rubus idaeus L.	raspberry ‘Lloyd George’ ‘Bulgarian rubin’, ‘Samodiva’, ‘Shopska alena’, ‘Iskra’ ‘Lyulin’	Micropropagation by applying LED lights; Adventitious shoot formation	Nacheva et al. (2021; 2023a); Stefanova et al. (2024); Georgieva et al. (2004a; 2004b; 2016)	FGI, BG; SU, BG; RIMSA, BG	20

	raspberry ’HimboTop‘	Micropropagation stages, liquid nutrient medium modification, bioreactors	Stanisavljević et al. (2018); Bošnjak et al. (2021)	UNIOS – FAZOS, HR	21
	raspberry ’Meeker’ ‘Willamete’	Adventitious organogenesis	Vujović et al. (2014)	FRI, RS	22
		Micropropagation (field trials; fruit volatile compounds; genetic fidelity; explant sterilization)	Leposavić et al. (2016); Popović et al. (2016); Vujović et al. (2017a); Anđelić et al. (2024)	FRI, RS	23
		Cold storage (shoots; artificial seeds)	Ružić et al. (2009b, 2011b)	FRI, RS	24
Rubus subg. *Rubus* Watson	blackberry ‘Čačanska Bestrna’	Adventitious regeneration (genetic fidelity; regeneration)	Vujović et al. (2010, 2014)	FRI, RS	25
		Micropropagation (PGRs; field trial; fruit volatile compounds; genetic fidelity; secondary metabolites of *in vitro* leaves and calli)	Ružić and Lazić (2006); Leposavić et al. (2016); Popović et al. (2016); Vujović et al. (2017a); Kolarević et al. (2021)	FRI, RS; AGRIF, RS	26
		Cold storage of artificial seeds	Ružić et al. (2011b)	FRI, RS	27
		Cryopreservation (encapsulation-dehydration; droplet vitrification)	Ružić and Vujović (2012); Vujović et al. (2011, 2015a)	FRI, RS	28
Vaccinium arctostaphylos L.	caucasian whorthleberry	Micropropagation	Nacheva and Kamburov, (2019)	FGI, BG	29

Vaccinium corymbosum L.	blueberry ‘Bluecrop’	Testing exposures to LED light	Nacheva et al. (2022)	FGI, BG	30
	blueberry	Vitrification; Stress conditions	Bošnjak (2022)	UNIOS – FAZOS, HR	31
	blueberry ‘Berkeley’, ‘Bluecrop’, ‘Goldtraube’	Micropropagation (medium composition)	Ružić et al. (2012a)	FRI, RS	32
	blueberry ‘Toro’	Cryopreservation (V and D cryo-plate)	Vujović et al. (2024a)	FRI, RS	33
Vaccinium vitis-idaea L.	lingonberry	Micropropagation	Georgieva et al. (2020; 2016)	RIMSA, BG	34
Vaccinium myrtillus L.	bilberry	Micropropagation	Georgieva et al. (2020; 2016)	RIMSA, BG	35
Vaccinium uliginosum L.	bog blueberry	Micropropagation	Georgieva et al. (2023)	RIMSA, BG	36
**Stone fruits**					
Olea europea L.	olive 'Chondrolia Chalkidikis' cv.	Micropropagation; Hyperhydricity reduction	Grigoriadou et al. (2002; 2007); Antonopoulou et al. (2012; 2018; 2020)	Vitro Hellas S.A., GR; AUTh, GR	37
	olive ‘Koroneiki’ cv.	Micropropagation	Roussos and Pontikis (2002); Ozkaya et al. (2003)	AUA, GR; MAICh, GR	38
Pittosporum angustifolium Lodd	weeping pittosporum	Micropropagation and acclimatization	Maniati and Papafotiou (2021)	AUA, GR	39
Prunus amygdalus x P. persica	’GF 677’ rootstock	Micropropagation	Spahiu and Sota (2009); Spahiu et al. (2015)	ATTC, AL	40

		In vitro rooting	Antonopoulou et al. (2005; 2007)	AUTh, GR	41
	'PR 204/84' rootstock	Micropropagation	Fotopoulos and Sotiropoulos (2005)	ELGO-DEMETRA, GR	42
Prunus armeniaca L.	apricot	Micropropagation	Bode et al. (2010)	ASA, AL	43
	apricot 'Bebecou' cv.	*In vitro* thermotherapy; Micropropagation	Koubouris et al (2007)	AUTh, GR	44
Prunus avium L	wild cherry	Micropropagation; Slow growth	Sota and Kongjika (2018)	Biotech-UT, AL	45
		Micropropagation	Scaltsoyiannes et al. (2009)	AUTh, GR	46
		Micropropagation (PGRs; PEG induced osmotic stress)	Vuksanović et al. (2020, 2022)	UNSFA, RS	47
		Micropropagation	Tančeva Crmarić and Kajba (2016)	UNiZG-FFWT, HR	48
	sweet cherry 'Lapins'	Micropropagation (PGRs; carbon sources)	Ružić and Vujović (2008); Ružić et al. (2008b)	FRI, RS	49
Prunus avium × *P. mahaleb*	'MxM' rootstock	Rhizogenesis	Sarropoulou et al. (2015b; 2017)	AUTh, GR	50
Prunus canescens Boiss	'Camil GM 79' rootstock	Micropropagation (medium composition)	Ružić et al. (2003, 2006)	FRI, RS	51
Prunus cerasifera Ehrh.	cherry plum	Micropropagation (PGRs)	Vujović et al. (2018b)	FRI, RS	52

		Cold storage	Ružić et al. (2012b, 2015b)	FRI, RS	53
		Cryopreservation (encapsulation -dehydration; V and D cryo-plate)	Ružić et al. (2015b); Vujović et al. (2015c)	FRI, RS	54
Prunus cerasus L.	sour cherry 'Oblačinska'	Thermotherapy and meristem culture	Jurković et al. (2008)	PIO, HR	55
		In vitro rooting	Mihaljević et al. (2013)	PIO, HR	56
		Photosynthetic variability under drought conditions	Viljevac Vuletić et al. (2022)	PIO, HR	57
		Micropropagation (explants collecting; PGRs)	Dorić et al. (2015); Vujović et al. (2024b)	UNSFA, RS;<br>FRI, RS	58
	D3 and D6 selections	Micropropagation (double-phase medium)	Dorić et al. (2014)	FRI, RS	59
	sour cherry 'Čačanski Rubin'	Adventitious regeneration (PGRs, subculturing; genetic fidelity)	Vujović et al. (2012a, 2013, 2014)	FRI, RS	60
	'Tabel Edabriz' rootstock	Micropropagation (carbon sources)	Ružić et al. (2008b)	FRI, RS	61
		Cold storage	Vujović et al. (2023a)	FRI, RS	62
Prunus cerasus × *P. canescens*	'Gisela 6' rootstock	Micropropagation	Papakosta and Sota (2023);	ATTC, AL	63

		Micropropagation	Nacheva and Gercheva (2008); Nikolova et al. (2021a)	FGI, BG	64
		Micropropagation (PGRs; prochloraz; double- phase medium; jasmonic acid)	Ružić et al. (2009a, 2015a); Vujović et al. (2009, 2012b); Dorić et al. (2014)	FRI, RS; UNSFA, RS	65
	‘Gisela 5’ rootstock	*In vitro* regeneration and rooting	Nacheva and Gercheva (2006; 2009)	FGI, BG	66
		Adventitious regeneration (genetic fidelity)	Vujović et al. (2012a, 2014)	FRI, RS	67
		Micropropagation (medium composition; explant sterilization; subculturing; PGRs)	Ružić et al. (2010, 2014a); Vujović et al. (2012b); Marjanović et al. (2019)	FRI, RS	68
		Cold storage	Ružić et al. (2015c); Vujović et al. (2023a)	FRI, RS	69
		Cryopreservation (droplet vitrification; vitrification; V and D cryo-plate)	Ružić et al. (2013, 2014b); Vujović et al. (2020c)	FRI, RS	70
	‘CAB-6P’, ‘Gisela 6’ and ‘Gisela 5’ rootstocks	Establishment of aseptic culture	Stanisavljević et al. (2017)	UNIOS – FAZOS, HR	71
		Establishment, multiplication and rooting	Bošnjak Mihovilović et al. (2012)	UNiZG – FAZ, HR	72

	'CAB-6P' and 'Gisela 6' rootstocks	Micropropagation	Sarropoulou et al. (2015a; 2016a; 2016b; 2017);	AUTh, GR	73
	'SL 64' rootstock	Micropropagation	Xilogiannis et al. (2008)	FITOTECHNIKI, GR	74
Prunus cerasifera × *P. persica*	'Krymsk 86' rootstock	Rhizogenesis induction	Tsafouros and Roussos (2021)	AUA, GR	75
Prunus domestica L.	plum 'Tropojane'	TIS bioreactors	Çuko et al. (2024)	Biotech-UT, AL	76
	plum 'Kyustendilska sinya'	Virus elimination	Nacheva et al. (2024); Milusheva et al. (2020)	FGI, BG	77
	plum 'Wangenheims' rootstock	Herbicides effect on *in vitro* conditions	Nacheva et al. (2012a)	FGI, BG	78
	plum 'Kyustendilska sinya' 'Valjevka'	*In vitro* virus-free plant production	Nacheva et al. (2002)	FGI, BG	79
	11 autochthonous plum genotypes	Micropropagation (explant sterilization; subculturing; PGRs)	Ružić et al. (2008c); Vujović et al. (2021a, 2022)	FRI, RS	80
		Cold storage	et al. (2012b); Vujović et al. (2020a)	FRI, RS	81
		Cryopreservation (droplet vitrification; V and D cryo-plate)	Vujović et al. (2015b, 2021c, 2023b)	FRI, RS	82
		Cryotherapy	Jevremović et al. (2023)	FRI, RS	83

	plum ‘Požegača’	Micropropagation (field trial)	Ružić and Cerović (2001)	FRI, RS	84
		Cold storage	Vujović et al. (2023a)	FRI, RS	85
		Cryopreservation (V and D cryo-plate)	Vujović et al. (2015c)	FRI, RS	86
	plum ‘Čačanska Lepotica’	Chemotherapy *in vitro*	Paunović et al. (2007)	FRI, RS	87
Prunus domestica subsp. *insititia*	‘Saint Julien’	Micropropagation by applying LED lights	Nacheva et al. (2023b)	FGI, BG	88
Prunus domestica × *P. cerasifera*	‘Docera 6’ rootstock	Micropropagation	Nacheva and Ivanova (2017)<br>Nikolova et al. (2021b)	FGI, BG	89
	‘Docera 6’ and ‘Dospina 235’ rootstocks	Micropropagation (PGRs)	Vujović et al. (2018b)	FRI, RS	90
Prunus fruticosa Pall.	SV1 and SV2 selections	Micropropagation (double-phase medium)	Dorić et al. (2014)	FRI, RS	91
Prunus fruticosa × *P. lannesiana*	‘Krymsk® 5’ rootstock	Optimization of micropropagation stages	Tsafouros and Roussos (2019; 2022; 2024)	AUA, GR	92
Prunus incisa x serrula	‘Inmil’ cherry rootstock	*In vitro* regeneration from protoplasts	Kondakova and Druart (2001)	ABI, BG	93
		Micropropagation (medium composition)	Ružić et al. (2001, 2006)	FRI RS	94
Prunus mahaleb L.	mahaleb cherry	Micropropagation; Slow growth	Sota and Kongjika (2011; 2014a; 2018)	Biotech-UT, AL	95

	M1 selection	Micropropagation (double-phase medium)	Dorić et al. (2014)	UNSFA, RS	96
Prunus salicina Lindley 'Methley' × *P. spinosa* L.	'Fereley Jaspi' rootstock	Micropropagation (explant sterilization; subculturing)	Ružić et al. (2010); Vujović et al. (2012b)	FRI, RS	97
Zizyphus jujuba Mill.	jujube	Micropropagation; Slow growth	Sota and Kongjika (2014b); Kongjika and Sota (2018)	Biotech-UT, AL; ASA, AL	98
**Pome fruits**					
Malus domestica Borkh.	apple 'Golden Delicious', 'Starking'	Micropropagation	Grazhdani et al. (2016); Cuko et al. (2017)	Biotech-UT, AL	99
	apple 'MM 106' rootstock	*In vitro* regeneration	Nacheva et al. (2009a); Gercheva et al. (2009)	FGI, BG	100
	apple 'Remo'	Virus elimination via chemotherapy	Nacheva and Milusheva (2008)	FGI, BG	101
	apple 'Topaz' cv.	*In vitro* propagation	Kereša et al. (2012)	UNiZG – FAZ, HR	102
	13 domesticated apple varieties	Establishment, proliferation	Batelja Lodeta et al. (2019)	UNiZG – FAZ, HR	103
	apple 'EM 9' rootstock	Oxidative stress; Micropropagation	Molassiotis et al. (2006)	AUTh, GR	104
	apple 'MM 106' rootstock	Optimizing micropropagation	Mouhtaridou et al. (2004); Sotiropoulos et al. (2006a)	ELGO-DEMETRA, GR	105

	apple ‘M 9’ rootstock	Optimizing micropropagation	Sotiropoulos et al. (2006a)	ELGO-DEMETRA, GR	106
	apple ‘M 4’ rootstock	Optimizing micropropagation	Sotiropoulos et al. (2007)	ELGO-DEMETRA, GR	107
	apple ‘Gala Must®’	Micropropagation; Cold storage	Ružić et al. (2016b)	FRI, RS	108
		Cryopreservation (droplet vitrification; vitrification)	Vujović et al. (2020b, 2021b)	FRI, RS	109
	apples ‘Melrose’, ‘Golden Delicious’, ‘Čadel’, ‘Gloster’	Adventitious organogenesis	Mitić et al. (2012)	IBISS, RS	110
		Micropropagation	Stanišić et al. (2018)	IBISS, RS	111
		Genetic transformation	Stanišić et al. (2019)	IBISS, RS	112
Malus sylvestris (L) Mill.	wild apple	Double-phase propagation system	Sota et al. (2021a)	Biotech-UT, AL	113
		ElecTIS bioreactor	Sota et al. (2021b)	Biotech-UT, AL	114
Pyrus communis L	‘OHF-333’ pear rootstock	Improvement of *in vitro* cultivation	Nacheva et al. (2009b); Dimitrova et. al. (2016; 2021b); Aleksandrova et al. (2021)	FGI, BG	115
		Optimizing micropropagation	Sotiropoulos et al. (2006b)	ELGO-DEMETRA, GR	116

	‘Pyrodwarf’ pear rootstock	Adventitious regeneration (genetic fidelity)	Vujović et al. (2014, 2018a)	FRI, RS	117
		Micropropagation (prochloraz; carbon sources; explant sterilization; PGRs; jasmonic acid)	Ružić et al. (2008a, 2008b, 2009a, 2010, 2011a, 2015a, 2016a); Vujović et al. (2018b)	FRI, RS	118
Pyrus pyraster L.	wild pear	Micropropagation; Slow growth	Kongjika and Sota (2018)	ASA, AL	119
		SETIS™ bioreactor	Sota et al. (2022)	Biotech-UT, AL	120
Pyrus spinosa Forskk (Syn: *P. amygdaliformis* Vill.)	almond-leaved pear	Micropropagation; Slow growth	Kongjika and Sota (2018)	ASA, AL	121
		Optimizing micropropagation	Tsoulpha et al. (2018); Alexandri et al. (2023)	AUTh, GR	122
**Nuts**					
Juglans regia L.	walnut	*In vitro* conservation; Synthetic seeds	Sota et al. (2023)	Biotech-UT, AL	123
		Oxidative stress avoidance Micropropagation	Myrselaj (Delija) et al. (2020; 2021)	Biotech-UT, AL	124
		Cytological evaluation of *in vitro* plants	Zekaj et al. (2003)	ASA, AL	125
	walnuts ‘Izvor 10’ ‘Lara’	Micropropagation, Effect of mT	Gandev et al. (2019); Nacheva and Gandev (2023)	FGI, BG	126

Pistacia terebinthus L.	turpentine tree	Micropropagation; *In vitro* conservation	Gercheva et al. (2008); Nacheva et al. (2012b; 2019)	FGI, BG	127
**Citrus fruits**					
Citrus aurantium L.	sour orange	*In vitro* rooting	Chatzissavvidis et al. (2009)	DUTH, GR	128
Citrus limon var. *limo*n (L.) Burm. f. x *C. latifolia* var. *latifolia*	hybrid	*In vitro* Thermotherapy, Meristem culture	Sarropoulou et al. (2024)	ELGO-DEMETRA, GR	129
Citrus maxima (Burm.) Merr.	pomelo	*In vitro* rooting	Papadakis et al. (2007)	AUTh, GR	130
Citrus unshiu (Mak.) Marc.	satsuma mandarin 'Zorica Rana Satsuma' cv.	Virus eradication, Micrografting	Hančević et al. (2009)	KRS, HR	131
Citrus sp.	Volkameriana, Citrumelo 'Swingle', Citrange 'Carrizo', *Poncirus trifoliata* 'Serra', *Poncirus trifoliata* 'Rubidoux' and 'Flying Dragon' rootstocks	*In vitro* propagation	Salis et al. (2017)	AUA, GR	132
	P. trifoliata, C. aurantium and Swingle citrumelo citrus rootstocks	*In vitro* propagation	Tzatzani et al. (2018)	AUTh, GR	133
	'Swingle Citrumelo', *C. taiwanica* and *C. aurantium* rootstocks	*In vitro* rooting	Dimassi et al. (2003)	AUTh, GR	134
Poncirus trifoliata L.	trifoliate orange	*In vitro* rooting	Chatzissavvidis et al. (2009)	DUTH, GR	135

**Ornamentals**					
Camptotheca acuminata Decne (Nyssaceae)	happy tree	Micropropagation	Nacheva et al. (2020)	FGI, BG	136
Ceratonia siliqua L.	carob	Micropropagation (leaf size; carbohydrate nutrition; anthocyanins)	Vinterhalter andVinterhalter (2003); Vinterhalter et al. (2001, 2007)	IBISS, RS	137
Chimonanthus praecox (L.) Link	wintersweet	Micropropagation (PGs; double-phase medium)	Kozomara et al. (2008)	IBISS, RS	138
Ebenus sibthorpii DC	Sibthorpe's ebony	Micropropagation	Bertsouklis et al. (2023b)	AUA, GR	139
Forsythia europaea Degen et Bald	Albanian forsythia	Micropropagation	Kongjika and Sota (2022)	ASA, AL	140
Magnolia grandiflora L.	magnolia	Micropropagation	Dimitrova et al. (2021a) Sokolov et al. (2015)	FGI, BG; IOP, BG	141
M. × soulangeana Soul.-Bod.	hybrid	Micropropagation	Dimitrova et al. (2021a) Sokolov et al. (2015)	FGI, BG; IOP, BG	142
Senna artemisioides (Gaudich. ex DC.) Randell	silver cassia	Micropropagation	Bertsouklis et al. (2023c)	AUA, GR	143
Tilia platyphyllos Scop.	large-leaved linden	Micropropagation	Ivanova et al. (2021)	FGI, BG	144
Tilia cordata Mill.	small-leaved linden	Micropropagation	Ivanova et al. (2021)	FGI, BG	145
Tilia tomentosa Moench.	silver linden	Micropropagation	Ivanova et al. (2021)	FGI, BG	146

Grape					
Vitis vinifera L	‘Shesh’, ‘Vlosh’	*In vitro* rhizogenezis	Kukali and Kongjika (2009)	ASA, AL	147
	18 Croatian autochthonous grapevine cultivars	Virus eradication	Marković et al. (2021)	UNiZG – FAZ, HR	148
	15 Croatian and international grapevine cultivars	Cryopreservation and cryotherapy	Marković et al. (2015)	UNiZG – FAZ, HR	149
	‘Agiorgitiko'	Virus-free plant production	Skiada et al. (2009)	AUTh, GR	150
	‘Malagouzia’, ‘Xinomavro’	Micropropagation	Skiada et al. (2010)	AUTh, GR	151
	'Giouroukiko', 'Serifiotiko'	Micropropagation	Kypraiou et al. (2019)	AUA, GR	152

*Only the institution of the first author of each paper is presented

Institutions’ acronyms: ABI, BG - Agrobioinstitute, Sofia, Agricultural Academy, Bulgaria; AGRIF, RS - University of Belgrade, Faculty of Agriculture, Zemun-Belgrade, Serbia; ASA, AL - Academy of Sciences of Albania; ATTC, AL - Agricultural Technology Transfer Center, Vlora, Albania; AUA, GR - Agricultural University of Athens, Greece; AUTh, GR - Aristotle University of Thessaloniki, Greece; BIOTECH-UT, AL - Department of Biotechnology, Faculty of Natural; Sciences, University of Tirana, Albania; DUTh, GR - Democritus University of Thrace, Greece; ELGO-DEMETRA, GR - ELGO-DEMETRA, Greece; FGI, BG - Fruit Growing Institute, Plovdiv, Agricultural Academy, Bulgaria; FRI, BG - Forest Research Institute, Sofia, Bulgarian Academy of Sciences, Bulgaria; FITOTECHNIKI, GR - FITOTECHNIKI, Greece; FRI, RS - Fruit Research Institute, Čačak, Serbia; IBISS, RS - Institute for Biological Research “Sinisa Stankovic”- National Institute of Republic of Serbia, University of Belgrade, Belgrade, Serbia; IM-BAS, BG - Institute of Microbiology, Plovdiv, Bulgarian Academy of Sciences; IOP, BG - Institute of Ornamental Plants, Negovan, Agricultural Academy, Sofia, Bulgaria; KRS, HR - Institute for Adriatic Crops and Karst Reclamation in Split, Croatia; MAICh, GR - Mediterranean Agronomic Institute of Chania, Greece; PIO, HR - Agricultural Institute Osijek, Croatia; RIMSA, BG - Research Institute of Mountain Stockbreeding and Agriculture, Troyan, Agricultural Academy, Bulgaria; SU - Sofia University, Bulgaria; UF, BG - University of Forestry, Sofia, Bulgaria; UNIOS-FAZOS, HR - Josip Juraj Strossmayer University of Osijek, Faculty of Agrobiotechnical Sciences, Croatia; UNiZG-FAZ, HR - University of Zagreb, Faculty of Agriculture, Croatia; UNiZG-FFWT, HR - University of Zagreb, Faculty of Forestry and Wood Technology, Croatia; UNSFA, RS - University of Novi Sad, Faculty of Agriculture, Novi Sad, Serbia.

TABLE 2 Scientific research on *in vitro* plant tissue culture of forest species in the Balkan countries (2001 – 2024)

Forest species	Common name /Cultivar/Genotype	Aim of the research	Reference	*Institution / Country	Row No. (R)
Acer platanoides L.	Norway maple	*In vitro* multiplication	Tomov and Iliev (2014)	UF, BG	1
Aesculus hippocastanum L.	horse chestnut	Androgenesis (abscisic acid; low temperature; activated charcoal; PEG; secondary embryogenesis; secondary metabolite)	Ćalić et al. (2003, 2005a, 2005b, 2012, 2013); Ćalić-Dragosavac et al., (2010a, 2010b, 2010c, 2011)	IBISS, RS	2
		Somatic embryogenesis (abscisic acid; shoot regeneration; rooting; shoot tip necrosis)	Ćalić et al. (2005a) Zdravković-Korać et al. (2012, 2022)	IBISS, RS	3
		Genetic transformation	Zdravković-Korać et al. (2003, 2004)	IBISS, RS	4
Aesculus flava Sol.	yellow buckeye	Androgenesis (anther and microspore culture)	Ćalić et al. (2005c); Zdravković-Korać et al. (2010, 2022)	IBISS, RS	5
		Somatic embryogenesis (stamen filament culture)	Zdravković-Korać et al. (2019, 2022)	IBISS, RS	6
Aesculus x carnea Zeyh.	red horse chestnut	Androgenesis (anther culture; culture medium)	Zdravković-Korać et al. (2022)	IBISS, RS	7

		Somatic embryogenesis	Zdravković-Korać et al. (2008; 2022)	IBISS, RS	8
Betula pendula Roth	silver birch	Adventitious shoot formation	Iliev and Tomita (2003); Iliev et al. (2001; 2003; 2010); Iliev (2017)	UF, BG	9
Frangula rupestris (Scop.) Schur.	rock buckthorn	Micropropagation (secondary metabolites)	Kovačević and Grubišić (2005)	FPUB, RS	10
Fraxinus excelsior L.	European ash	Adventitious shoot formation	Mitras et al. (2009); Dancheva et al. (2013); Dancheva and Iliev (2015)	UF, BG	11
Ginkgo biloba L.	ginkgo	*In vitro* multiplication	Nacheva and Ivanova (2017); Nacheva et al. (2017)	FGI, BG	12
Ilex aquifolium L.	holly	*In vitro* multiplication	Tsaktsira et al. (2018, 2021)	AUTh, GR	13
Paulownia elongata S.Y.Hu	empress tree	Micropropagation (PGRs)	Marković et al. (2013); Vujović et al. (2015d)	UB-FF, RS; FRI, RS	14
Paulownia elongata x fortunei	hybrid	Micropropagation from long-term preserved seeds	Gyuleva (2010)	FRI, BG	15
Pinus heldreichii H.Christ	Bosnian pine	Micropropagation (PGRs; physical factors)	Stojičić and Budimir (2004); Stojičić et al. (2007, 2008)	UB-FF, RS	16
		Somatic embryogenesis	Stojičić et al. (2018)	UNFSM, RS	17

Picea omorika (Pančic) Purk.	Serbian spruce	Somatic embryogenesis (PGRs)	Budimir (2003)	IBISS, RS	18
Pinus peuce Griseb.	Macedonian pine	Micropropagation (PGR; puls treatments)	Stojičić et al. (2012a, 2012b, 2024)	UNFSM, RS	19
Populus alba L	white poplar	*In vitro* regeneration	Tsvetkov et al. (2007c)	FRI, BG	20
		Vitrification for long-term storage	Tsvetkov et al. (2009)	FRI, BG	21
		Micropropagation (PGRs; silver ions)	Kovačević et al. (2010);	ILFE, RS	22
		In vitro selection (salinity; drought; acidity; lead tolerance; nickel)	Vuksanović et al. (2016, 2019a, 2019b, 2019c, 2019d, 2023); Kovačević et al. (2010, 2013, 2020); Katanić et al. (2008, 2015)	UNSFA, RS; ILFE, RS; IFVCNS, RS	23
Populus euphratica Oliv.	Euphrates poplar	Encapsulation; *In vitro* rooting	Tsvetkov et al. (2007a)	FRI, BG	24
Populus nigra L.	black poplar	*In vitro* selection (copper)	Vuksanović et al. (2017)	UNSFA, RS	25
Populus tremula L. × P. *tremuloides Mincx*	hybrid	Encapsulation; *In vitro* regeneration	Tsvetkov et al. (2006)	FRI, BG	26
		Vitrification for long-term storage	Tsvetkov et al. (2009)	FRI, BG	27
Quercus cerris L.	Turkey oak	Encapsulation; *In vitro* regeneration	Tsvetkov and Hausman (2005)	FRI, BG	28

Quercus robur L.	English oak	Encapsulation; *In vitro* regeneration	Tsvetkov and Hausman (2005)	FRI, BG	29
Quercus euboica Pap.	Euboea oak	Micropropagation	Kartsonas and Papafotiou (2007; 2009; 2010)	AUA, GR	30
Rhamnus catharticus L.	common buckthorn	Somatic embryogenesis (secondary metabolites)	Kovačević and Grubišić (2005)	IBISS, RS	31
Rhamnus fallax Boiss.	Carniolian buckthorn	Genetic transformation	Rosić et al. (2006)	IBISS, RS	32
Sorbus domestica L.	service tree	Encapsulation; *In vitro* rooting	Tsvetkov et al. (2007b)	FRI, BG	33

*Only the institution of the first author of each paper is presented

Institutions' acronyms: AU, BG - Agricultural University, Plovdiv, Bulgaria; AUA, GR - Agricultural University of Athens, Greece; AUTh, GR - Aristotle University of Thessaloniki, Greece; FGI, BG - Fruit Growing Institute, Plovdiv, Agricultural Academy, Bulgaria; FPUB, RS - University of Belgrade, Faculty of Pharmacy, Belgrade, Serbia; FRI, BG - Forest Research Institute, Sofia, Bulgarian Academy of Sciences, Bulgaria; FRI, RS - Fruit Research Institute, Čačak, Serbia; IBISS, RS - Institute for Biological Research "Sinisa Stankovic"- National Institute of Republic of Serbia, University of Belgrade, Belgrade, Serbia; IFVCNS, RS - Institute of Field and Vegetable Crops, Novi Sad, Serbia; ILFE, RS - Institute of Lowland Forestry and Environment, University of Novi Sad, Novi Sad, Serbia; UB-FF, RS - University of Belgrade, Faculty of Forestry, Belgrade, Serbia; UF, BG - University of Forestry, Sofia, Bulgaria; UNFSM, RS - University of Niš, Faculty of Sciences and Mathematics, Niš, Serbia; UNSFA, RS - University of Novi Sad, Faculty of Agriculture, Novi Sad, Serbia.

References

Aleksandrova, D., Dimitrova, N., Nikolova, V., and Nacheva, L. (2021). Floating system improves acclimatization of micropropagated pear plantlets. *Acta Hortic*. 1327, 727–732. doi: 10.17660/ActaHortic.2021.1327.97

Alexandri, S., Tsaktsira, M., Hatzilazarou, S., Kostas, S., Nianiou-Obeidat, I., Economou, A., et al. (2023). Selection for sustainable preservation through *in vitro* propagation of mature *Pyrus spinosa* genotypes rich in total phenolics and antioxidants. *Sustainability* 15, 4511. doi: 10.3390/su15054511

Anđelić, T., Vujović, T., Jevremović, D., Tomić, J., and Radivojević, D. (2024). Comparative study of different surface sterilization treatments and optimal month for establishment of aseptic cultures of raspberry cultivars. *J. Cent. Eur. Agric.* 25, 470–480. doi: 10.5513/JCEA01/25.2.4201

Antonopoulou, C., Dimassi, K., Chatzissavvidis, C., Papadakis, I., and Therios, I. (2012). The effect of explant type and nutrient medium on the *in vitro* proliferation of olive (*Olea europaea* L. "chondrolia chalkidikis"). *Acta Hortic*. 949, 185–189. doi: 10.17660/actahortic.2012.949.25

Antonopoulou, C., Dimassi, K., Therios, I., and Chatzissavvidis, C. (2018). Does dikegulac affect *in vitro* shoot proliferation and hyperhydricity incidence in olive explants? *Hortultural Sci*. 45, 125–130. doi: 10.17221/212/2016-HORTSCI

Antonopoulou, C., Dimassi, K., Therios, I., Chatzissavvidis, C., and Dichala, O. (2020). Comparative study of agar and vermiculite on micropropagation and hyperhydricity of olive (*Olea europaea* L.) explants. *Agric. Food* 8, 64–70.

Antonopoulou, C., Dimassi, K., Therios, I., Chatzissavvidis, C., and Papadakis, I. (2007). The effect of Fe-EDDHA and of ascorbic acid on *in vitro* rooting of the peach rootstock GF-677 explants. *Acta Physiologiae Plantarum* 29, 559–561. doi: 10.1007/s11738-007-0067-9

Antonopoulou, C., Dimassi, K., Therios, I., Chatzissavvidis, C., and Tsirakoglou, V. (2005). Inhibitory effects of riboflavin (Vitamin B2) on the *in vitro* rooting and nutrient concentration of explants of peach rootstock GF 677 (*Prunus amygdalus* × *P. persica*). *Scientia Hortic*. 106, 268–272. doi: 10.1016/j.scienta.2005.02.019

Bacu, A., Mata (Sota), V., and Kongjika, E. (2013). Pomegranates of Albania, the molecular evaluation of their genetic diversity and possible *in vitro* propagation of best varieties. *Agric. Forestry* 59, 157–165.

Bacu, A., Mata (Sota), V., Kongjika, E., Kukali, E., and Damiano, C. (2009). Interspecific variability of RAPDs and micropropagation of some pomegranate varieties (*Punica granatum* L.) of central and northwestern Albania. *Albanian J. Natural Tech. Sci. (AJNTS)* 24, 35–47.

Batelja Lodeta, K., Vujević B., Žanetić, M., Gugić, J., Očić, V., Škić Bobić, B., et al. (2019). Evaluation of morphological and chemical characteristics and micropropagation of traditionally grown domesticated apple varieties in Croatia. *J. Cent. Eur. Agric.* 20, 274–291. doi: 10.5513/JCEA01/20.1.2305

Bertsouklis, K., Naksi, K., and Aretaki, P. E. (2023c). *In vitro* germination and regeneration of Senna artemisioides, a valuable leguminous ornamental shrub. *Notulae Botanicae Horti Agrobotanici Cluj-Napoca* 51, 12992. doi: 10.15835/nbha51112992

Bertsouklis, K. F., and Papafotiou, M. (2009). *In vitro* propagation of *Arbutus andrachne* L. *Acta Hortic*. 813, 477–480. doi: 10.17660/actahortic.2009.8

Bertsouklis, K. F., and Papafotiou, M. (2011). Effect of various cytokinins on micropropagation of *Arbutus × andrachnoides* Link. *Acta Hortic*. 923, 213–218. doi: 10.17660/actahortic.2011.9

Bertsouklis, K., Paraskevopoulou, A. T., and Petraki, E. (2023a). *In vitro* regeneration from adult node explants of *Juniperus oxycedrus*. *Notulae Botanicae Horti Agrobotanici Cluj-Napoca* 51, 13062. doi: 10.15835/nbha51113062

Bertsouklis, K., Vazaka-Vodena, D., Bazanis, A. E., and Papafotiou, M. (2023b). Studies on seed germination and micropropagation of *Ebenus sibthorpii*, an endemic shrub of Greece with potential ornamental use. *Horticulturae* 9, 1300. doi: 10.3390/horticulturae9121300

Bode, D., Kongjika, E., and Sota, V. (2010). 'Tëdhëna paraprake të shumimit *in vitro* të species së egër autoktone *Prunus armeniaca* L. *Research/Kërkime* 18, 1–9.

Bošnjak, D. (2022). The optimization of blueberry micropropagation (*Vaccinium corymbosum* L.) using nanobiotechnology in liquid immersion (TIB/TIS) bioreactor system. Josip Juraj Strossmayer University of Osijek, Faculty of Agrobiotechnical Sciences Osijek, Osjek (Croatia.

Bošnjak, D., Marković, M., Agić, D., Vinković, T., Tkalec Kojić, M., Ravnjak, B., et al. (2021). The influence of nutrient media modification on the morphological parameters in raspberry (*Rubus idaeus* L.) micropropagation in the liquid and semi-solid media. *Poljoprivreda* 27, 22–29. doi: 10.18047/poljo.27.1.3

Bošnjak Mihovilović, A., Habuš Jerčić, I., Prebeg, T., Tomaz, I., Pavičić, A., Barić, M., et al. (2020). Light source and cytokinin type affect multiplication rate, chlorophyll content and stomata formation of *Amelanchier alnifolia* shoots *in vitro*. *J. Cent. Eur. Agric.* 21, 826–838. doi: 10.5513/JCEA01/21.4.2909

Bošnjak Mihovilović, A., Kereša, S., Jerčić, I. H., and Barić, M. (2012). The effect of cytokinin type and explant orientation on axillary shoot proliferation and *in vitro* rooting of 'Gisela 5'cherry rootstock. *J. Food Agric. Environ*. 10, 616–620.

Budimir, S. (2003). Developmental histology of organogenic and embryogenic tissue in. *Picea omorika* culture. *Biol. Plantarum* 47, 467–470. doi: 10.1023/B: BIOP.0000023898.83886.7d

Ćalić, D., Bohanec, B., Devrnja, N., Milojević, J., Tubić, Lj., Kostić, I., et al. (2013). Impact of abscisic acid in overcoming the problem of albinism in horse chestnut androgenic embryos. *Trees* 27, 755–762. doi: 10.1007/s00468-012-0830-4

Ćalić, D., Devrnja, N., Milojević, J., Kostić, I., Janošević, D., Budimir, S., et al. (2012). Abscisic acid effect on improving horse chestnut secondary somatic embryogenesis. *HortScience* 47, 1741–1744. doi: 10.21273/HORTSCI.47.12.1741

Ćalić, D., Zdravković-Korać, S., Guč-Šćekić, M., and Radojević, Lj. (2003). Efficient haploid induction in microspore suspension culture of *Aesculus hippocastanum* L. and karyotype analysis. *Biol. Plantarum* 47, 289–292. doi: 10.1023/B: BIOP.0000022268.34748.d8

Ćalić, D., Zdravković-Korać, S., and Pemac, D. (2005b). Effect of low temperature on germination of androgenic embryos of *Aesculus hippocastanum*. *Biol. Plantarum* 49, 431–433. doi: 10.1007/s10535-005-0022-9

Ćalić, D., Zdravković-Korać, S., and Radojević, Lj. (2005a). Secondary embryogenesis in androgenic embryo cultures of *Aesculus hippocastanum*. *Biol. Plantarum* 49, 435–438. doi: 10.1007/s10535-005-0023-8

Ćalić, D., Zdravković-Korać, S., and Radojević, Lj. (2005c). "Plant regeneration in anther culture of yellow buckeye (*Aesculus flava* Marshall)," in *Quality Enhancement of Plant Production Through Tissue Culture*, *Proceedings of the COST 843 Action, Stará Lesná, Slovakia,* 28 June–3 July 2005. Eds. G. Libiaková and A. Gajdošová (Institute of Plant Genetics and Biotechnology, Nitra, Slovakia), 183–185.

Ćalić-Dragosavac, D., Stević, S., and Zdravković-Korać, S. (2010a). Impact of genotype, age of tree and environmental temperature on androgenesis induction of *Aesculus hippocastanum* L. *Afr. J. Biotechnol*. 9, 4042–4049.

Ćalić-Dragosavac, D., Stević, S., Zdravković-Korać, S., Milojević, J., Cingel, A., and Vinterhalter, B. (2011). Secondary metabolite of horse chestnut *in vitro* culture. *Adv. Environ. Biol.* 5, 267–270.

Ćalić-Dragosavac, D., Zdravković-Korać, S., Bohanec, B., Radojević, Lj., Vinterhalter, B., Stevović, S., et al. (2010b). Effect of activated charcoal, abscisic acid and polyethylene glycol on maturation, germination and conversion of *Aesculus hippocastanum* androgenic embryos. *Afr. J. Biotechnol.* 9, 3786–3793.

Ćalić-Dragosavac, D., Zdravković-Korać, S., Šavikin-Fodulović, K., Radojević, Lj., and Vinterhalter, B. (2010c). Determination of aescin content in androgenic embryos and hairy root culture of *Aesculus hippocastanum* (Hippocastanaceae). *Pharm. Biol.* 48, 563–567. doi: 10.3109/13880200903204000

Chatzissavvidis, C., Antonopoulou, C., Papadakis, I., Therios, I., and Dimassi, K. (2009). Effects of NAA and vitamin B2 on *in vitro* rooting of *Citrus*. *Acta Agriculturae Scandinavica Section B —Soil Plant Sci.* 60, 189–192. doi: 10.1080/09064710902785300

Çuko, B., Shkëmbi, L., Kongjika, E., and Sota, V. (2024). "Overcoming challenges for micropropagation of *Prunus domestica* cv. Tropojane in various TIS bioreactor systems," in *Book of Proceedings of the 2nd Conference of Cost Action CA21157 – COPYTREE: "In vitro culture of woody crops: Problem solving by new approaches"*, Jūrmala, Latvia, 22–24 April 2024. 142–150.

Çuko, B., Sota, V., and Kongjika, E. (2017). Stabilisation of *in vitro* rhizogenesis of two apple (*Malus domestica* L.) cultivars during micropropagation. *Albanian J. Agric. Sci.*, 257–262.

Dancheva, D., and Iliev, I. (2015). Factors affecting adventitious shoot formation in *Fraxinus excelsior* L. *Propagation Ornamental Plants* 15, 10–20.

Dancheva, D., Iliev, I., and Iliev, N. (2013). *In vitro* propagation of *Fraxinus excelsior* L., Muzeul Olteniei Craiova. *Oltenia. Studii úi comunicări. ùtiinĠele Naturii* 29, 78–84.

Dimassi, K., Chouliaras, V., Diamantidis, G., and Therios, I. (2003). Effect of iron and auxins on peroxidase activity and rooting performance of three citrus rootstocks *in vitro*. *J. Plant Nutr.* 26, 1023–1034. doi: 10.1081/PLN-120020073

Dimitrova, N., Nacheva, L., and Berova, M. (2016). Effect of meta-Topolin on the shoot multiplication of pear rootstock OHF-333 (*Pyrus communis* L.). *Acta Scientiarum Polonorum - Hortorum Cultus* 15, 43–53.

Dimitrova, N., Nacheva, L., Ivanova, V., and Medkov, A. (2021a). Improvement of *in vitro* growth and rooting of *Magnolia grandiflora* L. and *Magnolia* × *soulangeana* Soul.-Bod. *Acta Horticulturae,* 1327, 349–360. doi: 10.17660/ActaHortic.2021.1327.47

Dimitrova, N., Nikolova, V., and Nacheva, L. (2021b). Effect of acclimatization in a floating system on photosynthetic ability of pear plants (*Pyrus communis* L. OHF 333). *Acta Horticulturae,* 1327, 753–762. doi: 10.17660/ActaHortic.2021.1327.101

Dorić, D., Ognjanov, V., Barać, G., Ljubojević, M., Pranjić, A., Dugalić, K., et al. (2015). Use of *in vitro* propagation of 'Oblačinska' sour cherry in rootstock breeding. *Turkish J. Biol.* 39, 8. doi: 10.3906/biy-1412-85

Dorić, D., Ognjanov, V., Ljubojević, M., Barać, G., Dulić, J., Pranjić, A., et al. (2014). Rapid propagation of sweet and sour cherry rootstocks. *Notulae Botanicae Horti Agrobotanici Cluj-Napoca* 42, 488–494. doi: 10.1583/nbha4229671

Fotopoulos, S., and Sotiropoulos, T. E. (2005). *In vitro* propagation of the PR 204/84 (*Prunus persica* × *P. amygdalus*) rootstock: Axillary shoot production and rhizogenesis. *New Z. J. Crop Hortic. Sci.* 33, 75–79. doi: 10.1080/01140671.2005.9514333

Gandev, S., Nikolova, V., Dimanov, D., Ivanov, P. T., and Dimitrov, A. V. (2019). Propagation of a local walnut cultivar 'Izvor 10' by *in vitro* techniques and hot callus method. *Acta Hortic.* 1259, 115–120. doi: 10.17660/ActaHortic.2019.1259.19

Georgieva, M., Badjakov, I., Dincheva, I., Yancheva, S., and Kondakova, V. (2016). *In vitro* propagation of wild Bulgarian small berry fruits (bilberry, lingonberry, raspberry and strawberry). *Bulgarian J. Agric. Sci.* 22, 46–51.

Georgieva, M., Djilianov, D., Kondakova, V., Boicheva, R., Konstantinova, T., and Parvanova, D. (2004a). Regeneration from leaf explants of Bulgarian raspberry cultivars and elites. *Biotechnol. Biotechnol. Equip*. 18, 8–14. doi: 10.1080/13102818.2004.10817080

Georgieva, M., Djilianov, D., Konstantinova, T., and Parvanova, D. (2004b). Screening of Bulgarian raspberry cultivars and elites for osmotic tolerance *in vitro*. *Biotechnol. Biotechnol. Equip.* 19, 95–98. doi: 10.1080/13102818.2004.10817093

Georgieva, M., Kondakova, V., Georgiev, D., Hristova, D., and Pavlov, A. (2023). Micropropagation of bog blueberry (*Vaccinium uliginosum* L.) distributed in Bulgaria. *Food Sci. Appl. Biotechnol*. 6, 127–133. doi: 10.30721/fsab2023.v6.i1.222

Georgieva, M., Georgiev, D., Hristova, D., Kondakova, V., Badjakov, I., and Pavlov, A. (2020). Impact of different concentrations of cytokinins /zeatin and 2-iP/ on *in vitro* propagation of wild species of genus *Vaccinium*. *Journal of Mountain Agriculture on the Balkans,* 23(3), 204–217.

Gercheva, P., Nacheva, L., and Dineva, V. (2009). The rate of shoot regeneration from apple (*Malus domestica* Borkh.) leaves depending on the *in vitro* culture conditions of the source plants. *Acta Hortic.* 825, 71–76. doi: 10.17660/ActaHortic.2009.825.7

Gercheva, P., Zhivondov, A., Nacheva, L., and Avanzato, D. (2008). Transexual forms of pistachio (*Pistacia terebinthus* L.) from Bulgaria – biotechnological approaches for preservation, multiplication and inclusion in selection programs. *Bulgarian J. Agric. Sci.* 14, 449–453.

Grazhdani, M., Sota, V., Çuko, B., and Kongjika, E. (2016). Micropropagation of some Albanian cultivars of *Malus domestica* Borkh. via direct organogenesis. *J. Natural Tech. Sci*. (JNTS) XXI, 77–88.

Grigoriadou, K., Eleftheriou, E. P., and Vasilakakis, M. (2007). Hidden hyperhydricity may be responsible for abnormal development and acclimatization problems of micropropagated olive plantlets: an anatomical leaf study. *Acta Hortic.* 748, 103–106. doi: 10.17660/actahortic.2007.748.10

Grigoriadou, K., Vasilakakis, M., and Eleftheriou, E. P. (2002). *In vitro* propagation of the Greek olive cultivar 'Chondrolia Chalkidikis'. *Plant Cell Tissue Organ Culture* 71, 47–54. doi: 10.1023/A:1016578614454

Gyuleva, V. (2010). Micropropagation of hybrid paulownia from long-term preserved seeds. *Silva Balcanica* 11, 45–58.

Hančević, K., Hartl Musinov, D., Černi, S., Rošin, J., Krajačić, M., Gatin, Ž., et al. (2009). The production of Citrus tristeza virus-free Zorica Rana, a Croatian selection of Satsuma mandarin. *J. Food Agric. Environ.* 7, 254–257.

Hatzilazarou, S., Grammatikos, H., Economou, A. S., Rifaki, N., and Ralli, P. (2003). Rooting *in vitro* and acclimatization of *Myrtus communis* microcuttings. *Acta Hortic.* 616, 259–264. doi: 10.17660/ActaHortic.2003.616.35

Iliev, I. (2017). Factors affecting the axillary and adventitious shoot formation in woody plants *in vitro*. *Acta Hortic.* 1155, 15–27. doi: 10.17660/ActaHortic.2017.1155.2

Iliev, I., Kitin, P., and Funada, R. (2001). Morphological and anatomical study on *in vitro* root formation of silver bitch (*Betula pendula* Roth). *Propagation Ornamental Plants* 1, 10–19.

Iliev, I., Rubos, A., Scaltsoyiannes, A., Nellas, H., and Kitin, P. (2003). Anatomical study of *in vitro* obtained fasciated shoots from *Betula pendula* Roth. *Acta Hortic*. 616, 481–484. doi: 10.17660/ActaHortic.2003.616.76

Iliev, I., Scaltsoyiannes, A., Tsaktsira, M., and Gajdosova, A. (2010). Micropropagation of *Betula pendula* Roth cultivars by adventitious shoot induction from leaf callus. *Acta Hortic*. 885, 161–173. doi: 10.17660/actahortic.2010.885.21

Iliev, I., and Tomita, M. (2003). Micropropagation of *Betula pendula* Roth. 'Fastigiata' by adventitious shoot regeneration from leaf callus. *Propagation Ornamental Plants* 3, 20–26.

Ivanova, V., Nacheva, L., and Panchev, V. (2021). Possibilities for application of *in vitro* techniques in propagation of species of the genus *Tilia* sp. *Bulgarian J. Agric. Sci*. 27, 103–110.

Jevremović, D., Vasilijević, B., Anđelić, T., and Vujović, T. (2023). Effect of D and V cryo-plate methods for plum pox virus eradication from two plum cultivars. *Plant Cell Tissue Organ Culture* 152, 529–538. doi: 10.1007/s11240-022-02425-y

Jurković, Z., Dugalić, K., Viljevac, M., Piližota, I., Vokurka, A., Puškar, B., et al. (2008). Preliminary report on the use of biotechnology in sweet and sour cherry research. *Acta Agronomica Hungarica* 56, 417–420. doi: 10.1556/AAgr.56.2008.4.6

Kartsonas, E., and Papafotiou, M. (2007). Mother plant age and seasonal influence on *in vitro* propagation of *Quercus euboica* Pap., an endemic, rare and endangered oak species of Greece. *Plant Cell Tissue Organ Culture* 90, 111–116. doi: 10.1007/s11240-007-9232-5

Kartsonas, E., and Papafotiou, M. (2009). Micropropagation of *Quercus euboica* Pap., a rare endemic oak species in Greece. *Acta Hortic*. 813, 485–490. doi: 10.17660/ActaHortic.2009.813.65

Kartsonas, E., and Papafotiou, M. (2010). Effect of culture vessels size and covering material on leaf morphological and anatomical characteristics of *Quercus euboica in vitro* plantlets. *Acta Hortic*. 885, 191–196. doi: 10.17660/ActaHortic.2010.885.24

Katanić, M., Kovačević, B., Đordević, B., Kebert, M., Pilipović, A., Klašnja, B., et al. (2015). Nickel phytoremediation potential of white poplar clones grown *in vitro*. *Romanian Biotechnol. Lett.* 20, 10085–10096.

Katanić, M., Pilipović, A., Orlović, S., Kovačević, B., and Pekeč, S. (2008). The influence of lead on the *in vitro* growth and concentration of photosynthetic pigments in shoots of the white poplar (*Populus alba*) clones. *Forestry J*. 54, 29–36.

Kereša, S., Bošnjak, A. M., Baric, M., Jercic, I. H., Sarcevic, H., and Bisko, A. (2012). Efficient axillary shoot proliferation and *in vitro* rooting of apple cv. 'Topaz'. *Notulae Botanicae Horti Agrobotanici Cluj-Napoca* 40, 113–118. doi: 10.15835/nbha4017211

Kolarević, T., Milinčić, D. D., Vujović, T., Gašić, U. M., Prokić, L., Kostić, A.Ž., et al. (2021). Phenolic compounds and antioxidant properties of field-grown and *in vitro* leaves, and calluses in blackberry and blueberry. *Horticulturae* 7, 420. doi: 10.3390/horticulturae7110420

Kondakova, V., and Druart, P. H. (2001). True-to type protoclones regeneration from mesophyll protoplasts of "Inmil" cherry rootstock (*Prunus incisa* × *serrula*). *Acta Hortic*. 560, 521–524. doi: 10.17660/ActaHortic.2001.560.106

Kongjika, E., Çaushi, E., Preçi, B., Zekaj, Zh., Çeko, A., Leventakis, N., et al. (2003). Estimation of some micropropagation methods for the introduction of Kiwiplant (*Actinidia* spp.) in Albania. *Albanian J. Natural Tech. Sci. (AJNTS)* VIII, 17–35.

Kongjika, E., and Sota, V. (2022). Micropropagazione e conservazione *in vitro* della specie endemica Forsythia europaea Degen et Bald. *Acta Italus Hortus* 30, 108–113.

Kongjika, E., Sota, V., and Damiano, C. (2018). Costituzione di collezioni *in vitro* di alcune importanti specie arboree "minori" da frutto. *Acta Italus Hortus* 21, 53–58.

Koubouris, G. C., Maliogka, V. I., Efthimiou, K., Katis, N. I., and Vasilakakis, M. D. (2007). Elimination of Plum pox virus through *in vitro* thermotherapy and shoot tip culture compared to conventional heat treatment in apricot cultivar Bebecou. *J. Gen. Plant Pathol*. 73, 370–373. doi: 10.1007/s10327-007-0028-6

Kovačević, N., and Grubišiš D. (2005). *In vitro* cultures of plants from the Rhamnaceae: Shoot propagation and anthraquinones production. *Pharm. Biol.* 43, 420–424. doi: 10.1080/13880200590963691

Kovačević, B., Miladinović, D., Orlović, S., Katanić, M., Kebert, M., and Kovinčić, J. (2013). Lead tolerance and accumulation in white poplar cultivated *in vitro*. *South-East Eur. Forestry* 4, 3–12. doi: 10.15177/seefor.13-01

Kovačević, B., Orlović, S., Rončević, S., and Miladinović, D. (2010). The effect of silver ion, 1-naphthalene acetic acid and 6-benzylaminopurine on micropropagation of “Fastigiate” tree shape variety *Populus alba* Cl. LBM. *Acta Hortic*. 885, 197–202. doi: 10.17660/ActaHortic.2010.885.25

Kozomara, B., Vinterhalter, B., Radojević, Lj., and Vinterhalter, D. (2008). *In vitro* propagation of *Chimonanthus praecox* (L.), a winter flowering ornamental shrub. *In Vitro Cell. Dev. Biol. – Plant* 44, 142–147. doi: 10.1007/s11627-008-9113-6

Krasteva, G., Teneva-Angelova, T., Badjakov, I., Dincheva, I., Pavlov, A., and Georgiev, V. (2023). “Effect of basal medium composition on biomass accumulation and exopolysaccharydes production by *Aronia melanocarpa* (Michx) Elliott suspension culture,” in *Proceedings of the V International Agricultural, Biological and Life Science Conference*, Edirne, Turkey, 18–20 September 2023. 450–456.

Kukali, E., and Kongjika, E. (2009). Efekti i disa fitorregullatorëve të rritjes në rrënjëzimin e disa kultivarëve të hardhisë. *Buletini i Shkencave Natyrore* 7, 128–135.

Kypraiou, S., Stavrakaki, M., Bouza, D., and Biniari, K. (2019). Effect of various culture media on *in vitro* propagation of grapevine cultivars 'Giouroukiko' and 'Serifiotiko' (*Vitis vinifera* L.). *Acta Hortic*. 1242, 561–566. doi: 10.17660/ActaHortic.2019.1242.82

Lala, Y., Meta, K., Çuko, B., Sota, V., Benelli, C., Lambardi, M., et al. (2024a). “Synthetic seeds conversion to plantlets in *Punica granatum* L. cv. Devedishe after short-term storage at 4°C,” in *Proceedings of the 2nd Conference of Cost Action CA21157 – COPYTREE: “In vitro culture of woody crops: Problem solving by new approaches*, Jūrmala, Latvia, 22–24 April 2024. 82–89.

Lala, Y., Meta, K., Kongjika, E., and Sota, V. (2024b). Evaluation of the regenerative potential of *Punica granatum* L cv. Devedishe apical shoots, after following vitrification and encapsulation-vitrification processes. *Albanian J. Natural Tech. Sci. (AJNTS)* XXIX, 17–30.

Lala (Bami), Y., Sota, V., and Kongjika, E. (2022). “Stabilization of an effective sterilization protocol for obtaining aseptic cultures of pomegranate (*Punica granatum* L) under *in vitro* conditions,” in *Proceedings of the 4th International Conference on Applied Biotechnology (4th ICAB)*, University of Ioannina, Greece, 20 October 2022. 157, ISBN: 978-960-233-276-4.

Leposavić, A., Ružić, Đ., Karaklajić-Stajić, Ž., Cerović, R., Vujović, T., Żurawicz, E., et al. (2016). Field performance of micropropagated *Rubus* species. *Acta Scientiarum Polonorum Hortorum Cultus* 15, 3–14.

Maniati, M., and Papafotiou, M. (2021). *In vitro* propagation and *ex vitro* acclimatization of *Pittosporum angustifolium*. *Acta Hortic*. 1327, 361–366. doi: 10.17660/ActaHortic.2021.1327.48

Marjanović, T., Vujović, T., Đorđević, M., and Vranić, D. (2019). Micropropagation of cherry rootstock Gisela 5. *J. Mountain Agric. Balkans* 22, 211–227.

Marković, Z., Preiner, D., Stupić, D., Andabaka, Ž., Šimon, S., Vončina, D., et al. (2015). Cryopreservation and cryotherapy of grapevine (*Vitis vinifera* L.). *VITIS-Journal Grapevine Res*. 54, 247–251. doi: 10.5073/vitis.2015.54.special-issue.247-251

Marković, M., Vilotić, D., and Popovic, M. (2013). Propagation of *Pawlovnia elongata* S. Y. HU by axilary shoots. *Propagation Ornamental Plants* 13, 73–77.

Marković, Z., Zrilić, A., Šikuten, I., Štambuk, P., Tomaz, I., Vončina, D., et al. (2021). Cultivar and phenological stage effects on the success of *in vitro* meristem culture and GLRaV-3 elimination of Croatian autochthonous grapevine cultivars. *Agronomy* 11, 1395. doi: 10.3390/agronomy11071395

Mereti, M., Grigoriadou, K., Levantakis, N., and Nanos, G. D. (2003). *In vitro* rooting of strawberry tree (*Arbutus unedo* L.) in medium solidified by peat - perlite mixture in combination with agar. *Acta Hortic.* 616, 207–210. doi: 10.17660/ActaHortic.2003.616.25

Mereti, M., Grigoriadou, K., and Nanos, G. D. (2002). Micropropagation of the strawberry tree, *Arbutus unedo* L. *Scientia Hortic*. 93, 143–148. doi: 10.1016/s0304-4238(01)00330

Mihaljević, I., Dugalić, K., Tomaš, V., Viljevac, M., Puškar, B., Čupić, T., et al. (2013). Influence of different carbon sources on *in vitro* rooting of sour cherry cv. Oblačinska. *Pomologia Croatica* 19, 23–36.

Mihaljević, I., Tomaš, V., Vuković, D., and Dugalić, K. (2019). Propagation of three Blue Honeysuckle (*Lonicera caerulea* L.) cultivars in *in vitro* culture. *Pomologia Croatica* 23, 41–48. doi: 10.33128/pc.23.1-2.4

Milusheva, S., Nacheva, L., Benova, E., Marinova, P., Dimitrova, N., and Georgieva-Hristeva, A. (2020). Experiments on Plum Pox Virus inactivation from micropropagated plum plants through non-thermal plasma treatment. *Plant Prot. Bull*. 60, 83–90. doi: 10.16955/bitkorb.653564

Mitić, N., Stanišić, M., Milojević, J., Ninković, S., and Miletić, R. (2012). Optimization of *in vitro* regeneration from leaf explants of apple cultivars Golden Delicious and Melrose. *HortScience* 47, 1117. doi: 10.21273/HORTSCI.47.8.1117

Mitras, D., Kitin, P., Iliev, I., Dancheva, D., Scaltsoyiannes, A., Tsaktsira, M., et al. (2009). *In vitro* propagation of *Fraxinus excelsior* L. by epicotyls. *J. Biol. Res*. 11, 37–48.

Molassiotis, A., Sotiropoulos, T., Tanou, G., Diamantidis, G., and Therios, I. (2006). Boron-induced oxidative damage and antioxidant and nucleolytic responses in shoot tips culture of the apple rootstock EM 9 (*Malus domestica* Borkh). *Environ. Exp. Bot.* 56, 54–62. doi: 10.1016/j.envexpbot.2005.01.002

Mouhtaridou, G. N., Sotiropoulos, T. E., Dimassi, K. N., and Therios, I. N. (2004). Effects of boron on growth, and chlorophyll and mineral contents of shoots of the apple rootstock MM 106 cultured *in vitro*. *Biol. Plantarum* 48, 617–619. doi: 10.1023/B: BIOP.0000047169.13304.67

Myrselaj, M., Sota, V., and Kongjika, E. (2020). Reducing oxidative stress on zygotic embryos of walnut (*Juglans regia* L.) under *in vitro* conditions by their pretreatment with ascorbic acid. *Eur. J. Biotechnol. Genet. Eng.* 7, 23–30.

Myrselaj (Delija), M., Sota, V., Bitri, I., and Kongjika, E. (2021). *In vitro* propagation of four native cultivars of *Juglans regia* L. in Albania using various types of cytokinins. *Acta Biologica Turcica* 34, 177–185.

Nacheva, L., Dimitrova, N., Ivanova, V., Cao, F., and Zhu, Z. (2020). Micropropagation of *Camptotheca acuminate* DECNE (Nyssaceae) – endangered ornamental and medicinal tree. Acta *Universitatis Agriculturae Silviculturae Mendelianae Brunensis* 68, 679–686. doi: 10.11118/actaun202068040679

Nacheva, L., Dimitrova, N., Koleva-Valkova, L., Stefanova, M., Ganeva, T., Nesheva, M., et al. (2023b). *In vitro* multiplication and rooting of plum rootstock 'Saint Julien' (*Prunus domestica* subsp. *insititia*) under fluorescent light and different led spectra. *Plants* 12, 2125. doi: 10.3390/plants12112125

Nacheva, L., Dimitrova, N., Koleva-Valkova, L., Tarakanov, I., and Vassilev, A. (2021). Effect of LED lighting on the growth of raspberry (*Rubus idaeus* L.) plants *in vitro*. *Agric. Sci.* 29, 129–140. doi: 10.22620/agrisci.2021.29.015

Nacheva, L., Dimitrova, N., Koleva-Valkova, L., Tarakanov, I., and Vassilev, A. (2023a). Effect of LED lighting on the rooting of micropropagated raspberry (*Rubus idaeus* L.) plants. *Acta Hortic*. 1359, 113–122. doi: 10.17660/actahortic.2023.1359.13

Nacheva, L., Dimitrova, N., and Vassilev, A. (2022). Response of *in vitro* cultivated highbush blueberry (*Vaccinium corymbosum* L.) to different LED lighting. *Acta Hortic*. 1337, 17–24. doi: 10.17660/actahortic.2022.1337

Nacheva, L., and Gandev, S. (2023). Could meta-Topoline improve the multiplication and rooting of micropropagated walnut plants? – a case study with 'Lara' (*Juglans regia* L.). *Acta Hortic*. 1359, 87–94. doi: 10.17660/ActaHortic.2023.1359.10

Nacheva, L., and Gercheva, P. (2006). The effect of auxin type and concentration on *in vitro* rooting of Gisela 5 (cherry dwarf rootstock). *J. Mountain Agric. Balkans* 9, 1309–1316.

Nacheva, L., and Gercheva, P. (2008). Micropropagation of the sweet cherry clonal rootstock Gisela 6 (*Prunus cerasus* × *Prunus canescens*). *J. Mountain Agric. Balkans* 11, 1569–1581.

Nacheva, L., and Gercheva, P. (2009). Micropropagation of Gisela 5 (cherry dwarf rootstock): the effect of the type and the concentration of the carbohydrates in the nutrient medium. *Acta Hortic*. 825, 261–268. doi: 10.17660/actahortic.2009.825.41

Nacheva, L., Gercheva, P., and Dzhuvinov, V. (2009b). Efficient shoot regeneration system from pear rootstock OHF–333 (*Pyrus communis* L.) leaves. *Acta Horic.* 839, 195–201. doi: 10.17660/ActaHortic.2009.839.23

Nacheva, L., Gercheva, P., Ivanova, V., and Ibrahim, O. (2017). Meta-topolin improves lateral bud proliferation in micropropagation of *Ginkgo biloba* L. *Acta Hortic.* 1155, 355–359. doi: 10.17660/ActaHortic.2017.1155.52

Nacheva, L., Gercheva, P., and Zhivondov, A. (2012b). Rooting of micropropagated transsexual *Pistacia terebinthus* L. plants from Bulgaria. *Acta Hortic*. 940, 275–281. doi: 10.17660/actahortic.2012.940.39

Nacheva, L., Gercheva, P., Zhivondov, A., and Malchev, S. (2019). Improvement of the rooting of micropropagated *Pistacia terebinthus* L. *Acta Hortic*. 1259, 143–148. doi: 10.17660/actahortic.2019.1259.23

Nacheva, L., and Ivanova, V. (2017). Silver nitrate and chlorhexidine gluconate – effective surface sterilization agents in disinfection procedures at initiation of woody shoot tip and embryo culture. *J. BioScience Biotechnol*. 6, 187–190.

Nacheva, L., Ivanova, K., and Milusheva, S. (2002). Elimination of PPV in plum cvs Kyustendilska sinya and Valjevka through *in vitro* Techniques. *Acta Hortic*. 577, 289–291. doi: 10.17660/ActaHortic.2002.577.49

Nacheva, L., and Kamburov, I. (2019). *In vitro* establishment and culture of Caucasian whorthleberry (*Vaccinium arctostaphylos* l.). *Acta Hortic*. 1259, 149–154. doi: 10.17660/actahortic.2019.1259.24

Nacheva, L., and Milusheva, S. (2008). Preliminary results of the effect of ribavirin on *in vitro* cultivated apple plants with the aim of eliminating some viruses. *J. Mountain Agric. Balkans* 11, 129–137.

Nacheva, L., Milusheva, S., Marinova, P., Dimitrova, N., and Benova, E. (2024). Cold atmospheric plasma (CAP) treatment of *in vitro* cultivated plum plantlets—a possible way to improve growth and inactivate plum pox virus (PPV). *Processes* 12, 1387. doi: 10.3390/pr12071387

Nacheva, L., Rankova, Z., and Gercheva, P. (2012a). Effect of some soil herbicides of the vegetative habits and pigment content of *Prunus domestica* 'Wangenheims' plum rootstock under *in vitro* conditions. *Bulgarian J. Agric. Sci*. 18, 583–588.

Nacheva, L., Zlatev, Z., and Ivanova, K. (2009a). Effect of sucrose level on the photosynthetic ability of *in vitro* cultivated apple rootstock MM 106. *Acta Hortic*. 839, 343–350. doi: 10.17660/ActaHortic.2009.839.44

Nikolova, V., Akova, V., Ivanov, P., and Dimitrov, A. (2021a). *In vitro* propagation of cherries rootstock Gisela 6. *J. Mountain Agric. Balkans* 24, 326–337.

Nikolova, V., Akova, V., Nesheva, M., and Malchev, S. (2021b). *In vitro* propagation of plum rootstock 'Docera 6'. *Fruit Growing Res*. 37, 131–135. doi: 10.33045/fgr.v37.2021.18

Ozkaya, M. T., Lionakis, S. M., and Vasilakakis, M. D. (2003). Factors affecting the *in vitro* regeneration and the rooting of defoliated cuttings of the olive cultivars 'Kalamon' and 'Koroneiki.'. *Biotechnol. Biotechnol. Equip*. 17, 70–76. doi: 10.1080/13102818.2003.10817061

Papadakis, I. E., Dimassi, K., Therios, I. N., Bosabalidis, A. M., and Sotiropoulos, T. (2007). Effects of Mn on anatomy, growth and carbohydrate content of adventitious roots in *Citrus maxima* (Burm.) Merr. shoot explants. *J. Biol. Res*. 8, 199–206.

Papafotiou, M., Bertsouklis, K. F., and Trigka, M. (2013a). Micropropagation of *Arbutus unedo*, *A. andrachne*, and their natural hybrid, *A*. × *andrachnoides* from seedling explants. *J. Hortic. Sci. Biotechnol*. 88, 768–775. doi: 10.1080/14620316.2013.11513037

Papafotiou, M., Trigka, M., and Bertsouklis, K. F. (2013b). *In vitro* propagation of *Arbutus* × *andrachnoides* Link. from seedling tissues. *Acta Hortic*. 990, 405–408. doi: 10.17660/actahortic.2013.9

Papakosta, E., and Sota, V. (2023). Effects of plant growth regulators on micropropagation of Gisela 6 (*Prunus cerasus* × *P. canescens*) cherry rootstock. *Albanian J. Natural Tech. Sci. (AJNTS)* XXVIII, 65–75.

Papakosta, E., Sota, V., and Kongjika, E. (2022). Micropropagazione *in vitro* del mirto (*Myrtus communis* L.) mediante tecnica convenzionale e coltura liquida con bioreattore ElecTIS. *Acta Italus Hortus* 30, 36–41.

Paunović, S., Ružić Đ., Vujović, T., Milenković, S., and Jevremović, D. (2007). *In vitro* production of Plum pox virus-free plums by chemotherapy with ribavirin. *Biotechnol. Biotechnol. Equip.* 21, 417–421. doi: 10.1080/13102818.2007.10817486

Peel, M. C., Finlayson, B. L., and McMahon, T. A. (2007). Updated world map of the Köppen-Geiger climate classification. *Hydrology Earth System Sci*. 11, 1633–1644. doi: 10.5194/hess-11-1633-2007

Popović, B., Mitrović, O., Leposavić, A., Ružić, Đ., Cerović, R., Vujović, T., et al. (2016). Volatile compounds of fruits of raspberry 'Meeker' and blackberry 'Čačanska Bestrna' propagated by standard techniques and by *in vitro* micropropagation. *Acta Hortic*. 1139, 645–650. doi: 10.17660/ActaHortic.2016.1139.111

Rosić, N., Momčilović, I., Kovačević, N., and Grubišić, D. (2006). Genetic transformation of *Rhamnus fallax* and hairy roots as a source of anthraquinones. *Biol. Plantarum* 50, 514–518. doi: 10.1007/s10535-006-0081-6

Roussos, P. A., and Pontikis, C. A. (2002). *In vitro* propagation of olive (*Olea europaea* L.) cv. Koroneiki. *Plant Growth Regul.* 37, 295–304. doi: 10.1023/A:1020824330589

Ružić, Đj., and Cerović, R. (2001). Field performance of micropropagated plum cv Požegača. *Acta Agriculturae Serbica* VI, 11, 3–9.

Ruzić, Đ., Cerović, R., and Ćulafić, Lj. (2006). The effect of inheriting factor on mineral nutrition of low vigorous sweet cherry rootstocks. *Acta Hortic.* 725, 385–389. doi: 10.17660/ActaHortic.2006.725.50

Ružić, Đ., Cerović, R., and Vujović, T. (2010). Establishment of aseptic culture *in vitro* for new vegetative rootstocks for cherry, pear and plum. *Voćarstvo* 44, 35–41.

Ružić, Đ., and Lazić, T. (2006). Micropropagation as means of rapid multiplication of newly developed blackberry and black currant cultivars. *Agriculturae Conspectus Scientificus* 71, 149–153.

Ružić, Đ., Lazić, T., and Cerović, R. (2008b). Micropropagation of some *Prunus* and *Pyrus* genotypes *in vitro* as affected by different carbon sources. *Acta Hortic.* 795, 413–418. doi: 10.17660/ActaHortic.2008.795.62

Ružić, Đ., Sarić, M., Cerović, R., and Ćulafić, Lj. (2001). Changes in macroelement content of the media and in sweet cherry Inmil GM 9 shoots during *in vitro* culture. *J. Hortic. Sci. Biotechnol*. 76, 295–299. doi: 10.1080/14620316.2001.11511366

Ružić, Đ., Sarić, M., Cerović, R., and Ćulafić, Lj. (2003). Contents of macroelements and growth of sweet cherry rootstock *in vitro*. *Biol. Plantarum* 47, 463–465. doi: 10.1023/B: BIOP.0000023897.84367.41

Ružić, Đ., and Vujović, T. (2008). The effects of cytokinin types and their concentration on *in vitro* multiplication of sweet cherry cv Lapins (*Prunus avium* L.). *Hortic. Sci*. 35, 12–21. doi: 10.17221/646-HORTSCI

Ružić, Đ., and Vujović, T. (2012). Cryopreservation *in vitro* of blackberry 'Čačanska Bestrna' shoot tips by encapsulation dehydration. *Acta Hortic*. 946, 55–60. doi: 10.17660/ActaHortic.2012.946.5

Ružić, Đ., Vujović, T., and Cerović, R. (2008c). Propagation of autochthonous plum type Sitnica (*Prunus domestica* L.) by micropropagation *in vitro*. *Voćarstvo* 42, 103–109.

Ružić, Đ., Vujović, T., and Cerović, R. (2009b). Short-term *in vitro* cold storage of raspberry shoots. *J. Mountain Agric. Balkans* 12, 883–899.

Ružić, Đ., Vujović, T., and Cerović, R. (2011b). *In vitro* propagation of blackberry and raspberry after cold storage of encapsulated shoot tips. *Acta Hortic*. 908, 275–282. doi: 10.17660/ActaHortic.2011.908.36

Ružić, Đ., Vujović, T., and Cerović, R. (2012b). *In vitro* preservation of autochthonous plum genotypes. *Bulgarian J. Agric. Sci.* 18, 55–62.

Ružić, Đ., Vujović, T., and Cerović, R. (2013). Cryopreservation of cherry rootstock Gisela 5 (*Prunus cerasus* × *Prunus canescens*) shoot tips by droplet-vitrification technique. *J. Hortic. Res*. 21, 79–85. doi: 10.2478/johr-2013-0025

Ružić, Đ., Vujović, T., and Cerović, R. (2014b). Cryopreservation of cherry rootstock Gisela 5 using vitrification procedure. *Hortic. Sci.* 41, 55–63. doi: 10.17221/234/2013-HORTSCI

Ružić, Đ., Vujović, T., and Cerović, R. (2015b). *In vitro* conservation of *Prunus cerasifera* Ehrh. by encapsulation dehydration and 'Cold storage' techniques. *Acta Hortic*. 1099, 587–594. doi: 10.17660/ActaHortic.2015.1099.71

Ružić, Đ., Vujović, T., and Cerović, R. (2015c). *In vitro* conservation of cherry rootstock Gisela 5. *Indian J. Traditional Knowledge* 14, 191–197.

Ružić, Đ., Vujović, T., and Cerović, R. (2016a). *In vitro* multiplication of semidwarfing pear rootstock 'Pyrodwarf' in relation to cytokinin types. *Acta Hortic*. 1139, 279–284. doi: 10.17660/ActaHortic.2016.1139.49

Ružić, Đ., Vujović, T., and Cerović, R. (2016b). *In vitro* konzervacija jabuke sorte Gala Must®. *Voćarstvo* 50, 71–81.

Ružić, Đ., Vujović, T., Cerović, R., and Đorđević, M. (2015a). Potential application of jasmonic acid in *in vitro* rooting of low vigorous pear and cherry rootstocks. *Acta Hortic*. 1099, 895–900. doi: 10.17660/ActaHortic.2015.1099.114

Ružić, Đ., Vujović, T., Cerović, R., and Kuzmanović, M. (2009a). The influence of imidazole fungicides on multiplication *in vitro* of low vigorous pear and cherry rootstocks. *Acta Hortic*. 839, 79–86. doi: 10.17660/ActaHortic.2009.839.7

Ružić, Đ., Vujović, T., Cerović, R., Libiakova, G., and Gajdosova, A. (2012a). Micropropagation *in vitro* of highbush blueberry (*Vaccinium corymbosum* L.). *J. Berry Res*. 2, 97–103. doi: 10.3233/JBR-2012-030

Ružić, Đ., Vujović, T., Cerović, R., and Vranić, D. (2014a). The effects of cytokinin types and their concentration on *in vitro* multiplication of sweet cherry rootstock Gisela 5. *Voćarstvo* 48, 105–115.

Ružić, Đ., Vujović, T., Milenković, S., Cerović, R., and Miletić, R. (2008a). The influence of Imidazole fungicides on multiplication *in vitro* of Pyrodwarf pear rootstock. *Aust. J. Crop Sci*. 1, 63–68.

Ružić, Đ., Vujović, T., Nikolić, D., and Cerović, R. (2011a). *In vitro* growth responses of the 'Pyrodwarf' pear rootstock to cytokinin types. *Romanian Biotechnol. Lett.* 16, 6630–6637.

Salis, C., Papadakis, I. E., Kintzios, S., and Hagidimitriou, M. (2017). *In vitro* propagation and assessment of genetic relationships of citrus rootstocks using ISSR molecular markers. *Notulae Botanicae Horti Agrobotanici Cluj-Napoca* 45, 383–391. doi: 10.15835/nbha45210900

Sarropoulou, V., Chatzissavvidis, C., Dimassi-Theriou, K., and Therios, I. (2016b). Effect of asparagine, cysteine, citrulline, and glutamine on *in vitro* rooting and biochemical constituents in cherry rootstocks. *Biol. Plantarum* 60, 1–12. doi: 10.1007/s10535-015-0562-6

Sarropoulou, V., Dimassi-Theriou, K., and Ioannis, Th. (2015a). Effect of sodium nitroprusside on micropropagation and biochemical parameters of CAB-6P and Gisela 6 cherry rootstocks. *Turkish J. Biol.* 39. doi: 10.3906/biy-1409-68

Sarropoulou, V., Dimassi-Theriou, K., and Therios, I. (2015b). Efects of exogenous indole-3-butyric acid and myo-inositol on *in vitro* rooting, vegetative growth and biochemical changes in leaves and roots in the sweet cherry rootstock MxM 14 using shoot tip explants. *Theor. Exp. Plant Physiol*. 27, 191–201. doi: 10.1007/s40626-015-0044-4

Sarropoulou, V., Dimassi-Theriou, K., and Therios, I. (2016a). Effect of the ethylene inhibitors silver nitrate, silver sulfate, and cobalt chloride on micropropagation and biochemical parameters in the cherryrootstocks CAB-6P and Gisela 6. *Turkish J. Biol.* 40. doi: 10.3906/biy-1505-92

Sarropoulou, V., Dimassi-Theriou, K., and Therios, I. (2017). Effects of the exogenous polyamines on micropropagation of cherry rootstocks. *Indian J. Plant Physiol*. 22, 227–239. doi: 10.1007/s40502-017-0289-9

Sarropoulou, V., Grigoriadou, K., Maliogka, V. I., Sassalou, C. L., and Ziogas, V. (2024). The elimination of viroids through *in vitro* thermotherapy and a meristem tip culture from a new limonime hybrid (*Citrus* × *limon* var. *limon* (L.) Burm. f. × *Citrus latifolia* var. *latifolia*). *BioTech* 13, 37. doi: 10.3390/biotech13030037

Scaltsoyiannes, A., Tsoulpha, P., Iliev, I., Theriou, K., Tsaktsira, M., Mitras, D., et al. (2009). Vegetative propagation of ornamental genotypes of *Prunus avium* L. *Propagation Ornamental Plants* 9, 198–206.

Skiada, F., Grigoriadou, K., and Eleftheriou, E. (2010). Micropropagation of *Vitis vinifera* L. cv. 'Malagouzia' and 'Xinomavro'. *Open Life Sci*. 5, 839–852. doi: 10.2478/s11535-010-0073-6

Skiada, F. G., Grigoriadou, K., Maliogka, V. I., Katis, N. I., and Eleftheriou, E. P. (2009). Elimination of grapevine leafroll-associated virus 1 and grapevine rupestris stem pitting-associated virus from grapevine cv. Agiorgitiko, and a micropropagation protocol for mass production of virus-free plantlets. *J. Plant Pathol*. 91, 177–184. doi: 10.4454/jpp.v91i1.639

Sokolov, R., Atanasova, B., and Iakimova, E. (2015). Influence of iron sources in the nutrient medium on *in vitro* shoot multiplication and rooting of Magnolia and Cherry plum. *J. Hortic. Res*. 23, 27–38. doi: 10.2478/johr-2015-0014

Sota, V., and Kongjika, E. (2010). *In vitro* conservation of some Albanian populations of *Myrtus communis* L. *Natura Montenegrina* 9, 553–565.

Sota, V., and Kongjika, E. (2011). *In vitro* rapid regeneration of plantlets of wild mahaleb cherry (P*runus mahaleb* L.). *Albanian J. Natural Tech. Sci. (AJNTS)* XVII, 135–146.

Sota, V., and Kongjika, E. (2014a). The effect of nutrient media in micropropagation and *in vitro* conservation of wild population of mahaleb cherry (*Prunus mahaleb* L.). *J. Microbiol. Biotechnol. Food Sci*. 3, 453–456.

Sota, V., and Kongjika, E. (2014b). Slow growth *in vitro* conservation of *Zizyphus jujuba* Mill. *Agric. Forestry* 60, 27–37.

Sota, V., Kongjika, E., and Damiano, C. (2018). Collezioni *in vitro* di alcune specie autoctone del genere *Prunus* in Albania. *Acta Italus Hortus* 21, 28–34.

Sota, V., Çuko, B., and Kongjika, E. (2020). Micropropagation of *Myrtus communis* L. and comparison of epidermal glandular trichomes characteristics between *in vivo* and *ex vitro* plantlets. *J. Environ. Prot. Ecol*. 21, 535–543.

Sota, V., Benelli, C., Çuko, B., and Kongjika, E. (2021a). Effective use of a double phase culture system and activated charcoal on *in vitro* propagation of *Malus sylvestris* (L) Mill. *Advances in Horticultural Science,* 35(4), 361–365. doi: 10.36253/ahsc-11825

Sota, V., Benelli, C., Çuko, B., Papakosta, E., Depaoli, C., Lambardi, M., and Kongjika, E. (2021b). Evaluation of ElecTIS bioreactor for the micropropagation of *Malus sylvestris* (L.) Mill., an important autochthonous species of Albania. *Horticultural Science,* 48(1), 12–21. doi: 10.17221/69/2020-HORTSCI

Sota, V., Çuko, B., Benelli, C., Kongjika, E., and Lambardi, M. (2022). Innovazione nella micropropagazione e nella conservazione *in vitro* di pero selvatico (*Pyrus pyraster* L.). *Acta Italus Hortus*, 30, 30–35.

Sota, V., Benelli, C., Myrselaj, M., Kongjika, E., and Gruda, N. S. (2023). Short-term conservation of *Juglans regia* L. via synthetic seed technology. *Horticulturae* 9, 1–13. doi: 10.3390/horticulturae905055

Sotiropoulos, T. E. (2007). Effect of NaCl and $CaCl_2$ on growth and contents of minerals, chlorophyll, proline and sugars in the apple rootstock M4 cultured *in vitro*. *Biol. Plantarum* 51, 177–180. doi: 10.1007/s10535-007-0035-7

Sotiropoulos, T. E., Almaliotis, D., Papadakis, I., Dimassi, K. N., and Therios, I. N. (2006b). Effects of different iron sources and concentrations on *in vitro* multiplication, rooting and nutritional status of the pear rootstock 'OHF 333'. *Eur. J. Hortic. Sci*. 71, 222–226.

Sotiropoulos, T. E., and Dimassi, K. N. (2004). Response to increasing rates of boron and NaCl on shoot proliferation and chemical composition of *in vitro* kiwifruit shoot cultures. *Plant Cell Tissue Organ Culture* 79, 285–289. doi: 10.1007/s11240-004-4609-1

Sotiropoulos, T. E., Molassiotis, A. N., Mouhtaridou, G. I., Papadakis, I., Dimassi, K. N., Therios, I. N., et al. (2006a). Sucrose and sorbitol effects on shoot growth and proliferation *in vitro*, nutritional status and peroxidase and catalase isoenzymes of M 9 and MM 106 apple (*Malus domestica* Borkh.) rootstocks. *Eur. J. Hortic. Sci*. 71, 114–119. doi: 10.1079/ejhs.2006/338654

Spahiu, E., Rama, P., and Hodaj, B. (2015). Callus induction and adventitious shoot regeneration from different explants of rootstocks GF-677 (*Prunus amygdalus* × *P. persica*). *Albanian J. Agric. Sci*. 14, 36–40.

Spahiu, E., and Sota, V. (2009). Mikroshumimi "*in vitro*" i nënshartesës GF 677 (pjeshkë × bajame). *Buletini i shkencave bujqësore* 2, 7–11.

Stanisavljević, A., Bošnjak, D., Štolfa, I., Vuković, R., Kujundžić, T., and Drenjančević, M. (2017). Sterilization of different explant types in micropropagation of CAB-6p and Gisela 6 cherry rootstock. *Poljoprivreda* 23, 31–37. doi: 10.18047/poljo.23.2.5

Stanisavljević, A., Štolfa, I., Popović, B., Bošnjak, D., Kujundžić, T., Viljanac, B., et al. (2018). "Acclimatization of raspberry plants from TIB system inoculated with Bradyrhizobium sp. and plant growth promoting bacteria (PGPR)," in *Proceedings of 53rd Croatian and 13th International Symposium on Agronomy*, Vodice, Croatia, 18–23 February. 530–534.

Stanišić, M., Ćosić, T., Savić, J., Krstić-Milošević, D., Mišić, D., Smigocki, A., et al. (2019). Hairy root culture as a valuable tool for allelopathic studies in apple. *Tree Physiol*. 39, 888–905. doi: 10.1093/treephys/tpz006

Stanišić, M., Ninković, S., Savić, J., Ćosić, T., and Mitić, N. (2018). The effects of blactam antibiotics and hygromycin B on *de novo* shoot organogenesis in apple cv. Golden Delicious. *Arch. Biol. Sci*. 70, 179–190. doi: 10.2298/ABS170731037S

Stefanova, M., Nacheva, N., Ganeva, T., and Dimitrova, N. (2024). LED lighting affects the biomass accumulation and leaf stomatal characteristics of raspberry (*Rubus idaeus* l.) *in vitro*. *J. Cent. Eur. Agric*. 25, 492–501. doi: 10.5513/jcea01/25.2.4232

Stojičić, D., and Budimir, S. (2004). Cytokinin-mediated axillary shoot formation in *Pinus heldreichii*. *Biol. Plantarum* 48, 477–479. doi: 10.1023/B: BIOP.0000041109.27037.21

Stojičić, D., Budimir, S., Čokeša, V., and Uzelac, B. (2024). Optimization of *in vitro* regeneration of *Pinus peuce* (Gris.). *Horticulturae* 10, 97. doi: 10.3390/horticulturae10010097

Stojičić, D., Janošević, D., Uzelac, B., and Budimir, S. (2008). Factors influencing germination and growth of isolated embryos of *Pinus heldreichii*. *Arch. Biol. Sci*. 60, 673–679. doi: 10.2298/ABS0804673S

Stojičić, D., Janošević, D., Uzelac, B., Čokeša, V., and Budimir, S. (2012a). *In vitro* zygotic embryo culture of *Pinus peuce* Gris: Optimization of culture conditions affecting germination and early seedling growth. *Arch. Biol. Sci*. 64, 503–509. doi: 10.2298/ABS1202503S

Stojičić, D., Janošević, D., Uzelac, B., Čokeša, V., and Budimir, S. (2012b). Micropropagation of *Pinus peuce*. *Biol. Plantarum* 56, 362–364. doi: 10.1007/s10535-012-0099-x

Stojičić, D., Uzelac, B., and Budimir, S. (2018). “Bosnian pine *Pinus heldreichii* Christ,” in *Step Wise Protocols for Somatic Embryogenesis of Important Woody Plants*. Eds. S. M. Jain and P. Gupta (Springer, Cham), 49–62.

Stojičić, D., Uzelac, B., Janošević, D., Ćulafić, Lj., and Budimir, S. (2007). Induction of somatic embryogenesis in *Pinus heldreichii* culture. *Arch. Biol. Sci*. 59, 199–202. doi: 10.2298/ABS0703199S

Tančeva Crmarić, O., and Kajba, D. (2016). Micropropagation of wild cherry (*Prunus avium* L.) from a clonal seed orchard. *Šumarski list* 140, 273–282. doi: 10.31298/sl.140.5-6.6

Tomov, V., and Iliev, I. (2014). *In vitro* multiplication of *Acer platanoides* L. *Muzeul olteniei Craiova*. *Oltenia*. *Studii şi comunicări. Ştiinţele naturii* 30, 44–54.

Tsafouros, A., and Roussos, P. A. (2019). First report of Krymsk® 5 (cv. VSL 2) cherry rootstock *in vitro* propagation: studying the effect of cytokinins, auxins and endogenous sugars. *Notulae Botanicae Horti Agrobotanici Cluj-Napoca* 47, 152–161. doi: 10.15835/nbha47111276

Tsafouros, A., and Roussos, P. (2021). 'Krymsk 86' stone fruit rootstock: high *in vitro* rooting potential even in absence of auxins. *Acta Hortic*. 1322, 187–192. doi: 10.17660/ActaHortic.2021.1322.27

Tsafouros, A., and Roussos, P. A. (2022). Dopamine, chlorogenic acid, and quinones as possible cofactors of increasing adventitious rooting potential of *in vitro* Krymsk 5 cherry rootstock explants. *Agronomy* 12, 1154. doi: 10.3390/agronomy12051154

Tsafouros, A., and Roussos, P. A. (2024). *In vitro* propagation of commercially used Krymsk 5® (*Prunus fruticosa* × *Prunus lannesiana*) cherry rootstock: impact of sugar types and ph levels. *Agriculture* 14, 120. doi: 10.3390/agriculture14010120

Tsaktsira, M., Alevropoulos, A., Tsoulpha, P., Scaltsoyiannes, A., and Iliev, I. (2018). Inter and intra-genetic variation on rooting ability of *Ilex aquifolium* L. varieties and cultivars. *Propagation Ornamental Plants* 18, 131–138.

Tsaktsira, M., Chavale, E., Kostas, S., Pipinis, E., Tsoulpha, P., Hatzilazarou, S., et al. (2021). Vegetative propagation and ISSR-based genetic identification of genotypes of *Ilex aquifolium* ‘Agrifoglio Commune’. *Sustainability* 13, 10345. doi: 10.3390/su131810345

Tsoulpha, P., Alexandri, S., and Tsaktsira, M. (2018). Critical factors affecting an efficient micropropagation protocol for *Pyrus spinosa* Forskk. *J. Appl. Horticulture* 20, 190–195. doi: 10.37855/jah.2018.v20i03.33

Tsvetkov, I., Benelli, C., Capuana, M., De Carlo, A., and Lambardi, M. (2009). Application of vitrification-derived cryotechniques for long-term storage of poplar and aspen (*Populus* spp.) germplasm. *Agric. Food Sci*. 18, 160–166. doi: 10.2137/145960609789267515

Tsvetkov, I., and Hausman, J. F. (2005). *In vitro* regeneration from alginate-encapsulated microcuttings of *Quercus* sp. *Scientia Hortic*. 103, 503–507. doi: 10.1016/j.scienta.2004.06.013

Tsvetkov, I., Hausman, J. F., and Jouve, L. (2007c). Thidiazuron-induced regeneration in root segments of white poplar (*P. alba* L.). *Bulgarian J. Agric. Sci*. 13, 623–626.

Tsvetkov, I., Jouve, L., and Hausman, J. F. (2006). Effect of alginate matrix composition on regrowth of *in vitro*-derived encapsulated apical microcuttings of hybrid aspen. *Biol. Plantarum* 50, 722–724. doi: 10.1007/s10535-006-0115-0

Tsvetkov, I., Jouve, L., Hoffmann, L., and Hausman, J. F. (2007a). The medium composition differentially affects regrowth characteristics in *in vitro*-derived encapsulated shoot tips of *Populus euphratica* oliv. *Propagation Ornamental Plants* 7, 180–183.

Tsvetkov, I., Jouve, L., Hoffmann, L., and Hausman, J. F. (2007b). Effect of auxins and alginate encapsulation on *in vitro* rooting of *Sorbus domestica*. *Belgian J. Bot.* 140, 151–156. doi: 10.2307/20794636

Tzatzani, T. T., Dimassi, K., and Therios, I. (2018). Organogenesis of citrus rootstocks using mature explants. *J. Agric. Environ. Sci*. 7, 10–15. doi: 10.15640/jaes.v7n1a2

Viljevac Vuletić, M., Horvat, D., Mihaljević, I., Dugalić, K., Šimić, D., Čupić, T., et al. (2022). Photosynthetic variability of Oblačinska sour cherry ecotypes under drought. *Plants* 11, 1764. doi: 10.3390/plants11131764

Vinterhalter, B., Ninković, S., Kozomara, B., and Vinterhalter, D. (2007). Carbohydrate nutrition and anthocyanin accumulation in light grown and etiolated shoot cultures of carob (*Ceratonia siliqua* L.). *Arch. Biol. Sci*. 59, 51–56. doi: 10.2298/ABS0701051V

Vinterhalter, D., and Vinterhalter, B. (2003). Effect of sucrose nutrition on the histological structure of carob shoot cultures. *Acta Biologica Slovenica* 46, 55–60. doi: 10.14720/abs.46.2.16675

Vinterhalter, B., Vinterhalter, D., and Nešković, M. (2001). Effect of irradiance, sugars, and nitrogen on leaf size of *in vitro* grown *Ceratonia siliqua* L. *Biol. Plantarum* 44, 185–188. doi: 10.1023/A:1010230821452

Vujović, T., Anđelić, T., Marković, Z., Gajdošovà, A., and Hunkovà, J. (2024a). Cryopreservation of highbush blueberry, strawberry, and saskatoon using V and D cryo-plate methods and monitoring of multiplication ability of regenerated shoots. *In Vitro Cell. Dev. Biology–Plant* 60, 85–97. doi: 10.1007/s11627-023-10399-5

Vujović, T., Anđelić, T., and Rilak, B. (2024b). "Micropropagation of 'Oblačinska' sour cherry: a step towards genetic uniformity and commercial viability," in *Proceedings of 6th International Scientific Conference Modern Trends in Agricultural Production, Rural Development and Environmental Protection*, Vrnjačka Banja, Serbia, 27–28 June 2024. 121–127 (Belgrade: The Balkans Scientific Center of the Russian Academy of Natural Sciences).

Vujović, T., Anđelić, T., and Ružić, Đ. (2023a). Evaluation of multiplication potential of cold stored shoot cultures of selected fruit genotypes. *Voćarstvo* 57, 37–46. doi: 10.18485/pomology.2023.57.215_216.4

Vujović, T., Anđelić, T., Vasilijević, B., Jevremović, D., and Engelmann, F. (2023b). Cryopreservation of indigenous plums and monitoring of multiplication and rooting capacity of shoots obtained from cryopreserved specimens. *Plants* 12, 3108. doi: 10.3390/plants12173108

Vujović, T., Cerović, R., and Ružić, Đ. (2012a). Ploidy level stability of adventitious shoots of sour cherry 'Čačanski Rubin' and Gisela 5 cherry rootstock. *Plant Cell Tissue Organ Culture* 111, 323–333. doi: 10.1007/s11240-012-0197-7

Vujović, T., Cerović, R., Ružić, Đ., and Marjanović, T. (2018a). An assessment of genetic integrity of *in vitro* shoots of Pyrodwarf pear rootstock. *J. Mountain Agric. Balkans* 21, 166–182.

Vujović, T., Chatelet, Ph., Ružić, Đ., and Engelmann, F. (2015c). Cryopreservation of *Prunus* sp. using aluminium cryo-plates. *Scientia Hortic*. 195, 173–182. doi: 10.1016/j.scienta.2015.09.016

Vujović, T., Jevremović, D., Anđelić, T., and Vasilijević, B. (2022). "Optimization of the protocol for *in vitro* propagation of autochthonous plum genotype 'Metlaš'," in *Proceedings of the XIII International Scientific Agriculture Symposium 'AGROSYM 2022'*, Jahorina, Bosnia and Herzegovina, 6–9 October 2022. 166–172 (Sarajevo, Bosnia and Herzegovina: Faculty of Agriculture and Food Sciences).

Vujović, T., Jevremović, D., Glišić, I. S., Milošević, N., and Anđelić, T. (2021a). *In vitro* culture establishment and shoot multiplication of eight autochthonous plum genotypes. *Acta Hortic*. 1322, 179–186. doi: 10.17660/ActaHortic.2021.1322.26

Vujović, T., Jevremović, D., Marjanović, T., and Glišić, I. (2020a). *In vitro* propagation and medium-term conservation of autochthonous plum cultivar 'Crvena Ranka'. *Acta Agriculturae Serbica* 25, 141–147. doi: 10.5937/AASer2050141V

Vujović, T., Jevremović, D., Marjanović, T., and Ružić, Đ. (2021c). Cryopreservation of Serbian autochthonous plum 'Crvena Ranka' using aluminium cryo-plates. *Genetika* 53, 283–294. doi: 10.2298/GENSR2101283V

Vujović, T., Marjanović, T., Ružić, Đ., and Glišić, I. (2018b). *In vitro* propagation of plum rootstocks. J. *Pomology* 52, 91–97.

Vujović, T., Ružić, Đ., and Cerović, R. (2012b). *In vitro* shoot multiplication as influenced by repeated subculturing of shoots of contemporary fruit rootstocks. *Hortic. Sci.* 39, 101–107. doi: 10.17221/208/2011-HORTSCI

Vujović, T., Ružić, Đ., and Cerović, R. (2012c). Improvement of *in vitro* micropropagation of black currant 'Čačanska Crna'. *Acta Hortic*. 946, 123–128. doi: 10.17660/ActaHortic.2012.946.17

Vujović, T., Ružić, Đ., and Cerović, R. (2013). Micropropagation of sour cherry 'Čačanski Rubin' (*Prunus cerasus* L.). *Voćarstvo* 47, 109–119.

Vujović, T., Ružić, Đ., and Cerović, R. (2014). Adventitious organogenesis via intermediate callus formation in representatives of *Prunus*, *Pyrus* and *Rubus* genera. *Romanian Biotechnol. Lett*. 19, 9297–9309.

Vujović, T., Ružić, Đ., and Cerović, R. (2015a). Optimization of droplet vitrification protocol for cryopreservation of *in vitro* grown blackberry shoot tip. *Acta Hortic*. 1099, 595–601. doi: 10.17660/ActaHortic.2015.1099.72

Vujović, T., Ružić, Đ., and Cerović, R. (2015b). Cryopreservation *in vitro* of autochthonous *Prunus* sp. by droplet-vitrification. *Biologia* 70, 1359–1365. doi: 10.1515/biolog-2015-0162

Vujović, T., Ružić, Đ., and Cerović, R. (2015d). Establishment/introduction of fast and efficient *in vitro* propagation system of *Paulownia elongata*. *Šumarstvo* 3/4, 33–42.

Vujović, T., Ružić, Đ., and Cerović, R. (2021b). Cryopreservation of apple shoot tips by vitrification and subsequent plant regeneration. *Acta Hortic*. 1308, 33–40. doi: 10.17660/ActaHortic.2021.1308.6

Vujović, T., Ružić, Đ., Cerović, R., Leposavić, A., Karaklajić Stajić, Ž., Mitrović, O., et al. (2017a). An assessment of the genetic integrity of micropropagated raspberry and blackberry plants. *Scientia Hortic*. 225, 454–461. doi: 10.1016/j.scienta.2017.07.020

Vujović, T., Ružić, Đ., Cerović, R., and Šurlan Momirović, G. (2010). Adventitious regeneration in blackberry (*Rubus fruticosus* L.) and assessment of genetic stability in regenerants. *Plant Growth Regul*. 61, 265–275. doi: 10.1007/s10725-010-9474-9

Vujović, T., Ružić, Đ., Marjanović, T., and Jevremović, D. (2020c). "Application of V and D cryo-plate methods for the cryopreservation of cherry rootstock Gisela 5," in *Proceedings of the XI International Scientific Agricultural Symposium 'Agrosym 2020'*, Jahorina, Bosnia and Herzegovina, Sarajevo, 6–9 October 2020. 62–68 (Saravejo, Bosnia and Herzegovina: Faculty of Agriculture and Food Sciences).

Vujović, T., Ružić, Đ., Vranić, D., and Marjanović, T. (2020b). Cryopreservation *in vitro* of apple shoot tips following droplet-vitrification. *Acta Hortic*. 1289, 1–8. doi: 10.17660/ActaHortic.2020.1289.1

Vujović, T., Sylvestre, I., Ružić, Đ., and Engelmann, F. (2011). Droplet-vitrification of apical shoot tips of *Rubus fruticosus* L. and *Prunus cerasifera* Ehrh. *Scientia Hortic*. 130, 222–228. doi: 10.1016/j.scienta.2011.06.049

Vuksanović, V., Kovačević, B., Katanić, M., and Orlović, S. (2017). *In vitro* evaluation of copper tolerance and accumulation in *Populus nigra*. *Arch. Biol. Sci*. 69, 679–687. doi: 10.2298/ABS170210014V

Vuksanović, V., Kovačević, B., Kebert, M., Katanić, M., Pavlović, L., Kesić, L., et al. (2019d). Clone specificity of white poplar (*Populus alba* L.) acidity tolerance *in vitro*. *Fresenius Environ. Bull*. 28, 8307–8313.

Vuksanović, V., Kovačević, B., Kebert, M., Milović, M., Kesić, L., Karaklić, V., et al. (2019c). *In vitro* modulation of antioxidant and physiological properties of white poplar induced by salinity. *Glasnik Šumarskog fakulteta* 120, 179–196.

Vuksanović, V., Kovačević, B., Kebert, M., Pavlović, L., Kesić, L., Čukanović, J., et al. (2023). *In vitro* selection of drought-tolerant white poplar clones based on antioxidant activities and osmoprotectant content. *Front. Plant Sci*. 14. doi: 10.3389/fpls.2023.1280794

Vuksanović, V., Kovačević, B., Kesić, L., Pavlović, L., Vaštag, E., and Kebert, M. (2020). Effect of IBA and TIBA on rhizogenesis of Wild cherry *in vitro*. *Topola* 206, 5–11. doi: 10.5937/topola2006005

Vuksanović, V., Kovačević, B., Orlović, S., Kebert, M., Katanić, M., and Pavlović, L. (2019b). "Effect of drought on growth and multiplication of white poplar genotypes in tissue culture," in *Proceedings of the X International Scientific Agriculture Symposium 'Agrosym 2019'*, Jahorina, Bosnia and Herzegovina, Sarajevo, 3–6 October 2019. 1936–1940 (Saravejo, Bosnia and Herzegovina: Faculty of Agriculture and Food Sciences).

Vuksanović, V., Kovačević, B., Orlović, S., Kebert, M., and Kovač, M. (2019a). The influence of drought on growth and development of white poplar shoots *in vitro*. *Topola/Poplar*, 13–18.

Vuksanović, V., Kovačević, B., Orlović, S., Miladinović, D., Katanić, M., and Kebert, M. (2016). "The effect of medium pH on white poplar shoots' growth *in vitro*," in *Proceedings of the VII International Scientific Agriculture Symposium 'Agrosym 2016'*, Jahorina, Bosnia and Herzegovina, Sarajevo, 6–9 October 2016. 2868–2873 (Saravejo, Bosnia and Herzegovina: Faculty of Agriculture and Food Sciences).

Vuksanović, V., Kovačević, B., Stojnić, S., Kebert, M., Kesić, L., Galović, V., et al. (2022). Variability of tolerance of Wild cherry clones to PEG-induced osmotic stress *in vitro*. *iForest* 15, 265–272. doi: 10.3832/ifor4033-015

Xilogiannis, C., Xilogiannis, A., and Mpalas, E. (2008). Micropropagation of two cherry rootstocks and their behavior in the nursery and the orchard. *Acta Hortic*. 795, 429–434. doi: 10.17660/ActaHortic.2008.795.65

Zdravković-Korać, S., Ćalić, D., Druart, P., and Radojević, Lj. (2003). The horse chestnut lines harboring the rol genes. *Biol. Plantarum* 47, 87–491. doi: 10.1023/B: BIOP.0000041051.81210.9b

Zdravković-Korać, S., Ćalić-Dragosavac, D., Milojević, J., Tubić, Lj., and Vinterhalter, B. (2010). "A comparison between anther culture and microspore suspension culture of Aesculus flava," in *Proceedings of the International Scientific Conference: Forest Ecosystems and Climate Changes*, Belgrade, Serbia, 9–10 March 2010, 49–54 (Belgrade: Institute of Forestry).

Zdravković-Korać, S., Ćalić-Dragosavac, D., Uzelac, B., Janošević, D., Budimir, S., Vinterhalter, B., et al. (2008). Secondary somatic embryogenesis versus caulogenesis from somatic embryos of *Aesculus carnea* Hayne: Developmental stage impact. *Plant Cell Tissue Organ Culture* 94, 225–231. doi: 10.1007/s11240-008-9399-4

Zdravković-Korać, S., Milojević, J., Belić, M., and Ćalić, D. (2022). Tissue culture response of ornamental and medicinal *Aesculus* species – A review. *Plants* 11, 277. doi: 10.3390/plants11030277

Zdravković-Korać, S., Muhovski, Y., Druart, P., Ćalić, D., and Radojević, Lj. (2004). *Agrobacterium rhizogenes*-mediated DNA transfer to *Aesculus hippocastanum* L. and the regeneration of transformed plants. *Plant Cell Rep*. 22, 698–704. doi: 10.1007/s00299-004-0756-4

Zdravković-Korać, S., Tubić, Lj., Devrnja, N., Ćalić, D., Milojević, J., Milić, M., et al. (2019). Somatic embryogenesis from stamen filaments of *Aesculus flava* Sol. And peroxidase activity during the transition from friable to embryogenic callus. *Scientia Hortic*. 247, 362–372. doi: 10.1016/j.scienta.2018.12.021

Zdravković-Korać, S., Tubić, Lj., Milojević, J., Devrnja, N., Kostić, I., and Ćalić, D. (2012). "Rooting and preventing shoot-tip necrosis of *in vitro* cultured horse chestnut shoots," in *Proceedings of the International Scientific Conference: Forest in Future–Sustainable Use, Risks and Challenges*, Belgrade, Serbia, 4–5 October 2012, 389–396 (Belgrade: Institute of Forestry).

Zekaj, Zh., Kongjika, E., Çaushi, E., and Preçi, B. (2003). Morphogenesis and histogenesis of walnut (*Juglans regia* L.) cultivated *in vitro*. *Albanian J. Natural Tech. Sci. (AJNTS)* VIII, 83–101.
